# A flexible hippocampal population code for experience relative to reward

**DOI:** 10.1038/s41593-025-01985-4

**Published:** 2025-06-11

**Authors:** Marielena Sosa, Mark H. Plitt, Lisa M. Giocomo

**Affiliations:** 1 Department of Neurobiology, Stanford University School of Medicine, Stanford, CA USA; 2 Howard Hughes Medical Institute, Stanford University School of Medicine, Stanford, CA USA; 3 Present Address: Department of Molecular and Cell Biology, University of California Berkeley, Berkeley, CA USA

**Keywords:** Hippocampus, Reward

## Abstract

To reinforce rewarding behaviors, events leading up to and following rewards must be remembered. Hippocampal place cell activity spans spatial and non-spatial episodes, but whether hippocampal activity encodes entire sequences of events relative to reward is unknown. Here, to test this possibility, we performed two-photon imaging of hippocampal CA1 as mice navigated virtual environments with changing hidden reward locations. We found that when the reward moved, a subpopulation of neurons updated their firing fields to the same relative position with respect to reward, constructing behavioral timescale sequences spanning the entire task. Over learning, this reward-relative representation became more robust as additional neurons were recruited, and changes in reward-relative firing often preceded behavioral adaptations following reward relocation. Concurrently, the spatial environment code was maintained through a parallel, dynamic subpopulation rather than through dedicated cell classes. These findings reveal how hippocampal ensembles flexibly encode multiple aspects of experience while amplifying behaviorally relevant information.

## Abstract (web summary)

Sosa et al. find that hippocampal neural activity in mice encodes both environmental location and experience relative to rewards, spanning distances far from reward, through parallel and flexible population-level codes.

## Main

Memories of positive experiences are essential for reinforcing rewarding behaviors and must be updated when knowledge of reward changes^1,2^. We questioned how the brain amplifies memories of events surrounding reward while maintaining a stable representation of the external world. The hippocampus provides a potential neural circuit for this process. Hippocampal place cells fire in one or few locations, defined as place fields^3^, and ‘remap’ across environments such that their place field locations or firing rates change^4–6^. Together, hippocampal neurons create behavioral timescale sequences of activity as an animal traverses space^4–7^ as well as in relation to non-spatial modalities, including time^8^ and multisensory decision-making variables^9–13^. This suggests the hippocampus encodes the progression of events as they unfold in a given experience^14–16^. Moreover, the hippocampus prioritizes coding for aspects of experience that are salient or relevant to the animal’s goals^1,2,9–14,16–18^. However, it remains unclear how the hippocampus encodes events relative to multiple aspects of experience while simultaneously amplifying those most relevant to behavior.

The presence of food or water reward is a salient event that is consistently prioritized. Place cells cluster near (that is, ‘over-represent’) rewarded locations^1,2,19–24^, with a small subpopulation active precisely at reward sites even when they are moved^22^. Furthermore, optogenetic activation of cells with place fields near reward drives reward-seeking actions^25^, suggesting a causal role in behavior. However, these prior studies focused on hippocampal representations highly proximal to rewards. To support memory of events leading up to and following reward, hippocampal activity encoding events distant from reward must also update when reward conditions change. Yet it remains unclear whether the hippocampus encodes such sequences of events around rewards separately from other stimuli.

We reasoned that reward may anchor hippocampal activity across the entire environment, creating a map for experience in reference to remembered rewards in parallel to a map for space. We speculated that previously reported reward-specific cells^22^ may comprise a subset of a larger population encoding an entire sequence of events relative to reward. This hypothesis predicts that moving the reward within a constant environment should induce predictable remapping even at locations far from the reward, preserving behavioral timescale firing order between neurons relative to each reward location. In parallel, another subpopulation should preserve their firing relative to the spatial environment, allowing the hippocampus to flexibly anchor to both the spatial and reward reference frames^4,12,17,26,27^ for solving the task at hand. Here, we found that the hippocampus indeed learned a generalized representation of the task anchored to reward, while also maintaining a spatial map in dissociable population codes.

## Results

### Monitoring neural activity during a reward learning task

**Fig. 1:**
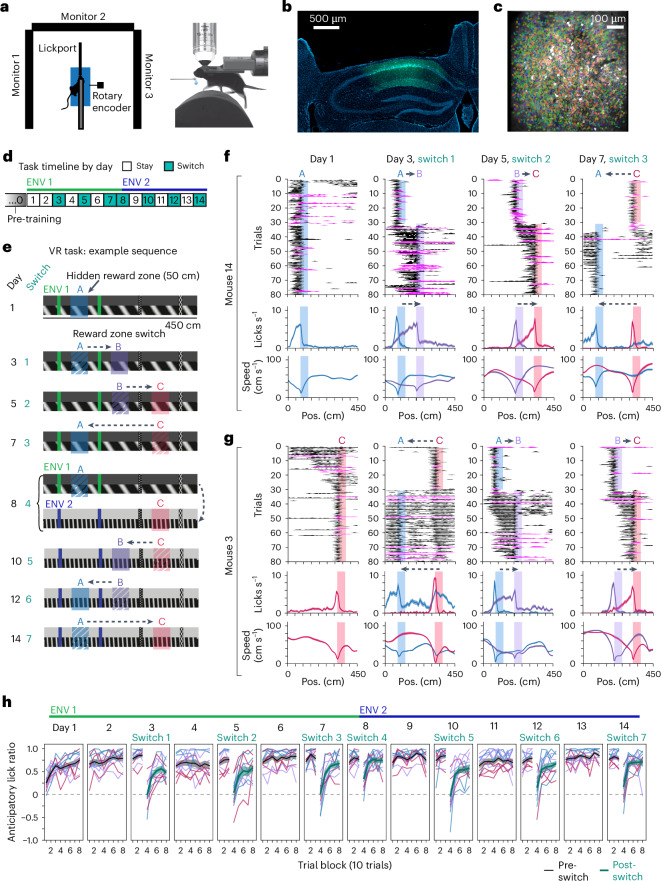
2P hippocampal imaging in a VR task with changing hidden reward locations. **a**, Top-down (left) and side (right) view of the head-fixed VR setup. Adapted from previous work^6^. A 5% sucrose water reward is delivered when the mouse licks a capacitive port. **b**, Coronal histology showing imaging cannula implantation site and calcium indicator GCaMP7f expression under the pan-neuronal synapsin promoter (AAV1-Syn-jGCaMP7f, green) in dorsal hippocampal area CA1 (DAPI, blue). **c**, Example field of view (mean image) from the same mouse in **b** (mouse m12). Identified neurons in shaded colors (*n* = 1,780 cells). **d**, Task timeline. Reward remains at the same location on ‘stay’ days, and moves to a new location after the first 30 trials on ‘switch’ days (~80 trials per session). Colored bars indicate an example environment (ENV) order (*n* = 9 mice experienced ENV 1 then ENV 2; *n* = 2 mice experienced ENV 2 then ENV 1). **e**, Side views of virtual linear tracks showing an example reward switch sequence (reward zone switch order was counterbalanced across animals). Shaded regions illustrate hidden reward zones. **f**, Example behavior (mouse m14) on day 1 and first three switches. Top, smoothed lick rasters as a function of position (pos.). Rewarded trials, black; omission trials, magenta. Shaded regions, active reward zone. In **f** and **g**, data are presented as mean ± s.e.m. lick rate (middle row) or mean ± s.e.m. running speed (bottom row) for trials at each reward location (A, blue; B, purple; C, red). **g**, Same as **f**, but for an example mouse (m3) that experienced a different reward zone switch sequence. See Extended Data Fig. 1d for all sequence allocations. **h**, Behavior across animals performing reward switches (*n* = 11 mice). An anticipatory lick ratio of 1 indicates licking exclusively in the 50 cm before a reward zone (‘anticipatory zone’; Extended Data Fig. 1e) and 0 (horizontal dashed line) indicates chance licking across the whole track outside of the reward zone. Colored bars, example environment order as shown in **d**. Thin lines, individual mice; colored by the active reward zone for each mouse on a given set of trials (A, blue; B, purple; C, red). Black and teal lines show mean ± s.e.m. across mice for pre-switch and post-switch trials, respectively. Source data

**Extended Data Fig. 1:**
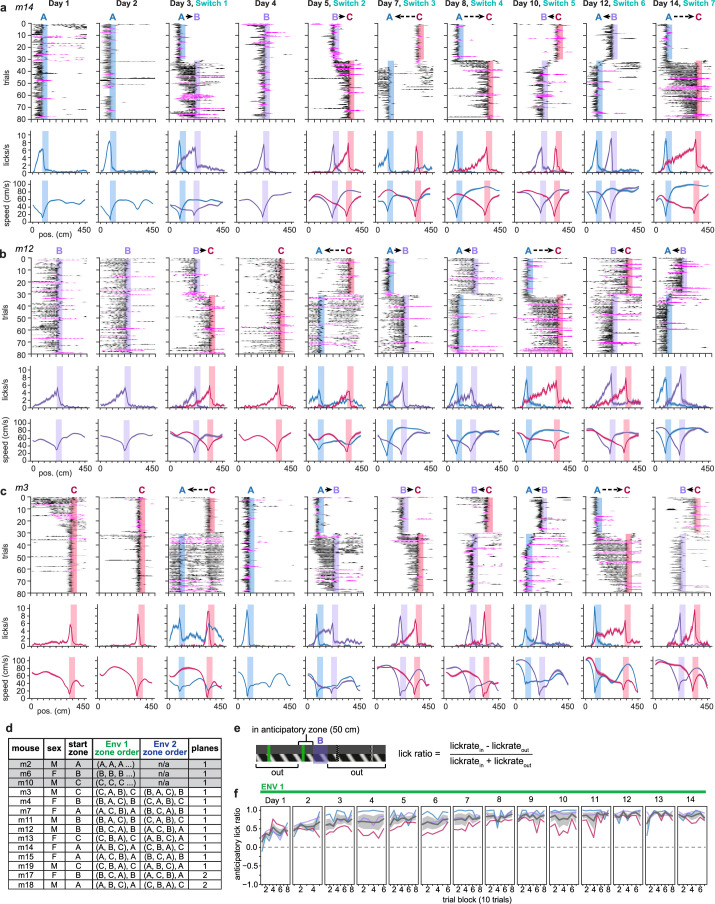
Behavior across multiple reward switch sequences. **a**) Expanded behavior over the 14-day task for the example animal in Fig. 1f (m14). Top: smoothed lick raster over 80 trials per session; black, rewarded trials; magenta, omission trials. Shaded regions indicate the reward zone active on each set of trials. In (**a**–**c**), middle row: mean ± SEM lick rate; bottom row: mean ± SEM running speed, across trials at each reward location (blue = reward zone A, purple = B, red = C). **b**) Behavior as in (**a**) for an example animal that started the task with reward zone “B” (m12). **c**) Expanded behavior as in (**a**) for the example animal in Fig. 1g (m3). **d**) Mouse identities, sexes, order of reward zone switches, and planes imaged in the superficial-deep axis of CA1. Top 3 mice (gray highlight) are “fixed-condition” mice that maintained the same reward zone and environment throughout the experiment, bottom 11 mice are “switch” mice. Reward zone orders are shown as “(0, 1, 2), 0” where 0 is the first reward zone in a given environment, 1 is the first switch (2nd zone), 2 is the second switch (3rd zone), and a return to zone 0 is the third switch. Note that reward zones are listed chronologically, but for m17 and m18 the environment order was swapped. **e**) Schematic depicting anticipatory lick ratio calculation around reward zone “B” as an example. The anticipatory lick ratio compares lick rate in an anticipatory zone 50 cm before the start of the reward zone vs. everywhere outside the reward zone (excluding the reward zone to eliminate consummatory licks). A value of 1 indicates licking exclusively in the anticipatory zone, 0 indicates chance licking everywhere outside the reward zone. **f**) Anticipatory lick ratio across blocks of 10 trials for fixed-condition animals (n = 3 mice). Colored lines indicate individual mice and the reward zone active for each mouse (blue = A, purple = B, red = C); gray lines and shading show mean ± SEM across mice.

To dissociate reward from other sensory stimuli and observe how hippocampal activity related to rewards evolves with experience, we performed two-photon (2P) calcium imaging of CA1 neurons expressing GCaMP7f in head-fixed mice learning a virtual reality (VR) navigation task (Fig. 1a–c). VR provided tight control over sensory stimuli and the animal’s trajectory, allowing us to observe neuronal remapping in relation to distant rewards, a phenomenon potentially less visible in freely moving scenarios. Furthermore, the task included multiple updates to a hidden reward zone across two environments, allowing us to dissociate spatially driven and reward-driven remapping. Imaging began on day 1 of task acquisition, in which animals traversed a unidirectional 450 cm virtual linear track (for example, Environment 1 (ENV 1)) with a hidden 50 cm reward zone where sucrose water was delivered operantly for licking (Fig. 1d,e). On day 3, the reward zone was moved within a ‘switch’ session to a new track location, signaled by automatic reward delivery on the first ten post-switch trials only if the mouse failed to lick in the new zone. On day 5, the reward zone was moved to a third location, then returned to its original location on day 7. On day 8, the reward switch coincided with the introduction of a novel environment (for example, ENV 2), in which the switch order was then reversed (Fig. 1e). On ‘stay’ days, the last reward location from the previous day was maintained. A separate ‘fixed-condition’ group of mice (*n* = 3) experienced only one reward location in a single environment (ENV 1) (Extended Data Fig. 1), to control for the effect of experience. To maintain engagement, the reward was randomly omitted on ~15% of trials. At the end of the track, mice passed through a variable length gray ‘teleport’ zone (~50 cm + temporal jitter up to 10 s; Methods) before starting the next lap.

Mice developed an anticipatory ramp of licking up to the reward zone start, accompanied by a compensatory decrease in running speed (Fig. 1f,g and Extended Data Fig. 1a–c), demonstrating successful task acquisition. After a reward switch, mice entered an exploratory period of licking before refining their licking to anticipate each new zone, typically within a session (Fig. 1f,g and Extended Data Fig. 1a–c). We quantified this improvement as the ratio of anticipatory lick rate 50 cm before the rewarded zone compared to outside this zone (Extended Data Fig. 1e). All mice improved and retained precise licking preceding the new reward zone (Fig. 1h; *n* = 11 mice), demonstrating accurate spatial learning and memory.

### Moving reward induced remapping spanning the environment

**Fig. 2:**
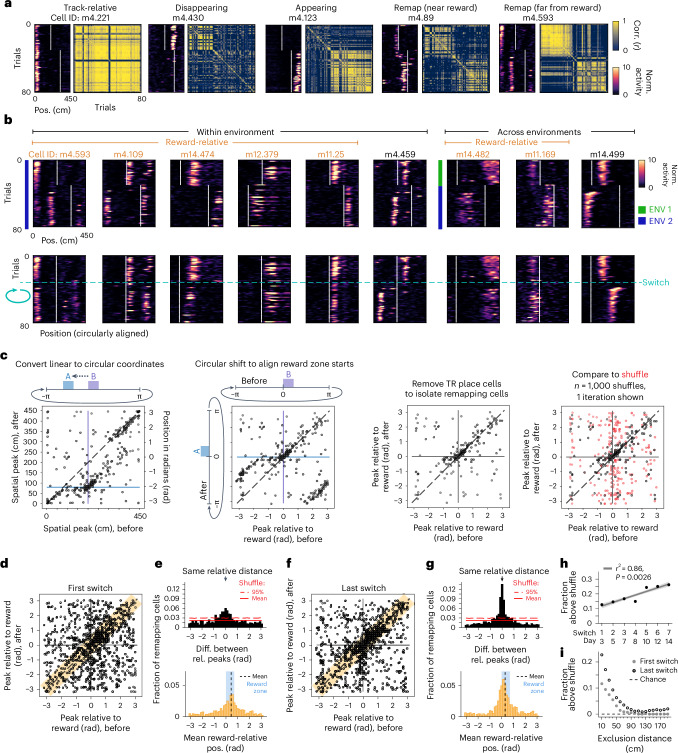
A subpopulation of CA1 cells remaps relative to reward. **a**, Five example cells (mouse m4, ENV 2, day 14). Left panel: spatial activity per trial normalized (norm.) to the cell’s mean activity. White lines, reward zone starts. Right panel: trial-by-trial correlation (corr.) matrix. **b**, Example RR cells (orange labels) versus non-RR remapping cells (black labels) (four mice, replicated in *n* = 11 mice; see Extended Data Figs. 2 and 4). Top: mean-normalized spatial activity, original track axis. Bottom: trials after the switch (blue line) circularly shifted to align reward locations within (day 14) and across environments (day 8). **c**, Left: example peak activity per place cell (translucent dots, jittered for visualization; m12, day 14) before versus after the switch in original track coordinates. Middle-left: data rotated in periodic coordinates to align the starts of reward zones at zero. Middle-right: TR cells removed. Right: data compared to a random-remapping shuffle (red). **d**, Peak activity per remapping cell relative to reward (day 3, *n* = 1,082 cells, 11 mice). Orange shading, ≤50 cm (~0.698 radians) between relative peaks. **e**, Top: histogram of the circular difference (diff). between relative (rel.) peaks of remapping cells after minus before the switch (orthogonal to the unity line in **d**). Solid and dashed red lines, mean and upper 95% of the shuffle distribution. Bottom: distribution of mean RR firing position for cells with ≤50 cm between relative peaks (orange shading in **d**; *n* = 453 cells, 11 mice, non-zero mean, 0.441 radians; 95% confidence interval (lower, upper), (0.306, 0.575); circular mean test). Black dashed line, mean; blue shading, reward zone. **f**, Same as **d** but for the last switch (day 14, *n* = 1,153 cells, 11 mice). **g**, Same as **e** but for the last switch (day 14). Bottom: *n* = 645 cells with ≤50 cm between relative peaks (non-zero mean, 0.292 radians; 95% confidence interval, (0.205, 0.380); circular mean test). **h**, Above-chance fraction of RR remapping cells grows linearly with task experience. Dots, combined fraction across *n* = 11 mice; gray line and shading, best fit ± s.d. of linear regression. **i**, Above-chance fraction of RR remapping cells after exclusion of cells within each distance (±) from both reward zone starts. Source data

**Extended Data Fig. 2:**
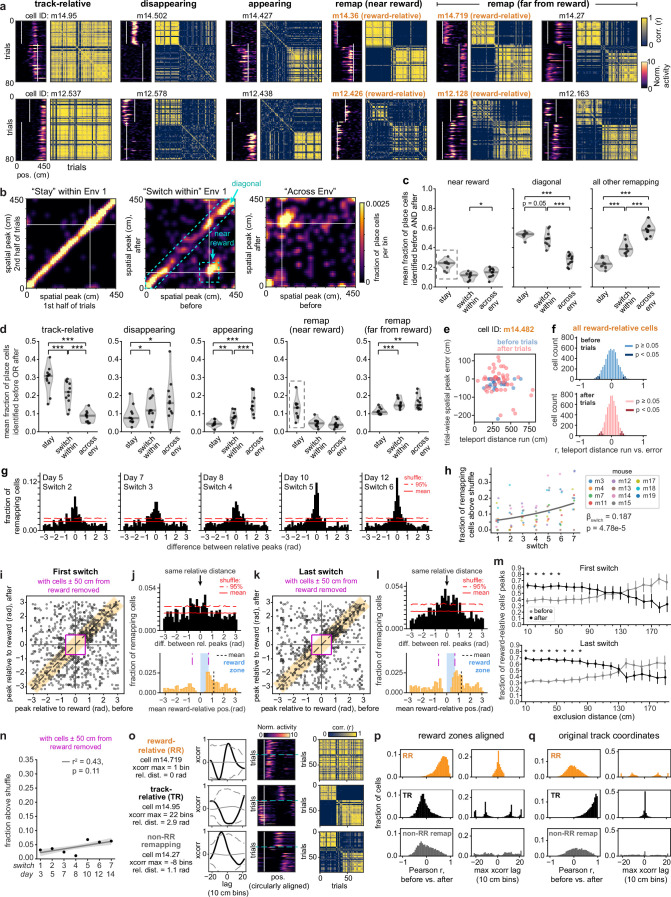
Quantification of remapping types and controls for reward-relative remapping. **a**) Example cells from 2 additional mice (top: m14, bottom: m12; Env 2, day 14), replicating the co-existence of remapping types within animal shown in Fig. 2a. Orange labels, “reward-relative” cells. **b**) Example smoothed 2D histograms of the fraction of place cells in mouse m14 with peak firing in each 10 cm bin before vs. after the reward zone switch (or in the 1st vs. 2nd half of trials on “stay” days), for a “stay” day (day 6, same reward location, same environment), “switch within” day (day 7, change reward location, same environment), or “across environment” day (day 8, change reward location, change environment). White lines, reward zone starts. Only cells with significant spatial information both before and after are included. A strong density along the diagonal on the “stay” day indicates place cells that remain track-relative throughout the session. On switch days, this diagonal degrades as more cells remap, and the off-diagonal band around the reward intersection appears (see also Fig. 2c). When the environment changes, the diagonal degrades further indicating more global remapping, but the reward-relative band is maintained. Cyan boxes, bins used for the “diagonal” and “near reward” categories shown in (**c**). **c**) Quantification of remapping using bins of the histograms illustrated in (**b**) (unsmoothed for quantification). Each dot is the mean fraction of cells across days of each type per animal (n = 11 mice); horizontal lines, median across mice. In (**c**, **d**), *p < 0.05, **p < 0.01, ***p < 0.001 from pairwise, paired two-sided t-tests between categories (“stay”, “switch within”, “across env”), performed on the logit-transformed fractions, Holm-corrected for multiple comparisons within subpanel. “Near reward”: cells with peaks ≤50 cm from both reward zone starts; gray dashed box: fraction of total place cells near the reward on stay days across the 1st to 2nd half of the session, thus no t-tests are shown for this category as the reward is not moved. Switch within vs. across env: t = −2.9, p = 0.015. “Diagonal”: peaks within ≤50 cm of the same linear position before vs. after. Stay vs. switch within: t = 2.2, p = 0.05; stay vs. across env: t = 14.6, p = 1.3e-7; switch within vs. across env: t = 10.7, p = 1.8e-6. “All other remapping”: all bins not near-reward and not along the diagonal. Stay vs. switch within: t = −9.1, p = 7.3e-6; stay vs. across env: t = −27.6, p = 2.7e-10; switch within vs. across env: t = −8.6, p = 7.3e-6. **d**) Mean fractions of place cells (across days of each type per animal) defined as track-relative, disappearing, appearing, remap near reward, or remap far from reward (see Methods), agnostic of which cells can be described as “reward-relative” since that remapping category cannot be defined on stay days. Track-relative: stay vs. switch within: t = 5.6, p = 2.2e-4; stay vs. across env: t = 23.4, p = 1.4e-9; switch within vs. across env: t = 9.1, p = 7.7e-6. Disappearing: stay vs. switch within: t = −3.4, p = 0.022; stay vs. across env: t = −2.8, p = 0.035; switch within vs. across env: t = −1.3, p = 0.23. Appearing: stay vs. switch within: t = −4.1, p = 0.0022; stay vs. across env: t = −9.6, p = 7.0e-6; switch within vs. across env: t = −5.5, p = 5.2e-4. Remap near reward: switch within vs. across env: t = 0.86, p = 0.41. As in (**c**), gray dashed box: fraction near reward on stay days without a reward switch, thus no statistical comparisons are shown. Remap far from reward: stay vs. switch within: t = −6.7, p = 1.5e-4; stay vs. across env: t = −4.6, p = 0.0020; switch within vs. across env: t = −0.67, p = 0.52. **e**) To test whether reward-relative neurons encode distance traveled since the last reward, we asked whether distance run in the variable length teleport period predicted an offset in the cell’s peak spatial firing (error from its mean) on the subsequent trial. Example reward-relative cell (cell m14.482 on day 8, switch 4; also shown in Fig. 2b), with no significant Spearman correlation between the teleport distance run and signed spatial peak error for either set of trials (dots). Blue, trials before the switch: r = 0.10, p = 0.59; Pink, trials after the switch: r = −0.14, p = 0.34. **f**) Spearman correlation coefficients between teleport distance run and spatial peak error on the subsequent trial, for all reward-relative cells (n = 5979 cells, 11 mice, 7 switch days). Histograms are stacked; dark shades, significant cells (p < 0.05): 6.7% of reward-relative cells on before trials, 7.5% on after trials. Light shades, non-significant cells: 93.3% of reward-relative cells on before trials, 92.5% on after trials. **g**) Histograms of the circular difference between relative peaks after minus before the switch for remapping place cells on switch days not shown in Fig. 2e, g (n = 11 mice; day 5 n = 997 cells, day 7 n = 1128 cells, day 8 n = 1157 cells, day 10 n = 1022 cells, day 12 n = 934 cells). **h**) Increase in above-chance reward-relative remapping across experience, at the level of individual animals. Each point, fraction of cells (logit-transformed) exceeding the “random-remapping” shuffle calculated within each mouse and switch day (n = 11 mice). Regression coefficient β and p-value (two-sided Wald test) are from a linear mixed effects model with switch index as the fixed effect and mice as random effects. Gray line, model prediction. **i**–**l**) Control for Fig. 2d–g, excluding cells with peaks ≤50 cm (~0.698 radians) from both reward zone starts (a 100-cm span, indicated by magenta lines). (**i**) n = 888 cells, 11 mice. (**k**) n = 795 cells, 11 mice. (**j**, top) and (**l**, top): The fraction of cells remapping relative to reward at distances greater than ±50 cm still exceeds the shuffle. (**j**, bottom) and (**l**, bottom): distribution of mean reward-relative positions for the cells in the orange shaded region around the unity line in (**i**) and (**k**), respectively. (**j**) significant non-zero mean = 1.145, 95% confidence interval [lower, upper]=[0.822, 1.468], circular mean test, n = 285 cells. (**l**) significant non-zero mean = 1.245, 95% confidence interval [lower, upper] = [0.931, 1.559], circular mean test, n = 303 cells. Note that cells firing within 50 cm of one reward but not the other may have a circular mean relative distance of <0.698 radians (<50 cm), visible as the small fractions between the magenta lines. **m**) Fraction of reward-relative cells (mean ± SEM across mice) with peak firing before (gray) or after (black) the reward zone start, following exclusion of cells within iteratively larger regions on either side of reward (that is, an exclusion distance of 10 cm is a 20 cm span around the reward zone start). *p < 0.00263 significance level with Bonferroni correction, two-sided z-test for proportions compared to a null hypothesis of a 50/50% split: First switch: 10 cm: p = 1.2e-17, 20 cm: p = 5.4e-12, 30 cm: p = 5.2e-7, 40 cm: p = 7.9e-7, 50 cm: p = 0.00014; Last switch: 10 cm: p = 5.4e-35, 20 cm: p = 1.0e-20, 30 cm: p = 1.1e-15, 40 cm: p = 3.9e-13, 50 cm: p = 3.1e-9, 60 cm: p = 3.4e-8, 70 cm: p = 9.3e-6, 80 cm: p = 0.000256. **n**) After excluding cells within 50 cm of the reward zone start, the fraction of reward-relative remapping cells above the shuffle shows a trending but non-significant increase across task experience. Gray line and shading, best fit ± SD of linear regression. **o**) Illustration of cross-correlation criterion to identify reward-relative (RR) (top row) vs. track-relative (TR) (middle row) vs. non-reward-relative (non-RR) remapping cells (bottom row). Each cell is also shown in (**a**, top row). Left column: cross-correlation (xcorr) between trial-averaged spatial firing with reward zones circularly aligned (middle column), before vs. after the switch. Gray lines, mean (solid) and 95% confidence interval (dashed) of the shuffle per cell. Listed at left: offset of the xcorr maximum above the shuffle, distance between relative peaks in radians. Teal line, reward zone switch trial. Right column: trial-by-trial correlation matrix using reward-aligned activity. Note the uniform structure for the RR cell vs. the block-like matrices for the TR and non-RR remapping cell, opposite of how these matrices appear in original linear track coordinates (**a**, top row). **p**) Left: Two-sided Pearson correlation coefficients for each subpopulation across 11 mice and 7 switch days, between each cell’s trial-averaged activity maps pre- vs. post-switch in reward-aligned coordinates. Two-sided Wilcoxon rank-sum tests: RR vs. TR: Z = 91.7, p < 1e-24 (n = 5979 RR cells, 7027 TR cells); RR vs. non-RR remapping: Z = 71.6, p < 1-e24 (n = 5979 RR cells, 4314 non-RR cells). Right: Maximum xcorr offsets above the shuffle for each subpopulation, with the RR distribution thresholded at ±5 bins. **q**) Same as (**p**), but using trial-averaged activity of each subpopulation in original linear track coordinates. Two-sided Wilcoxon rank-sum tests: TR vs. RR: Z = 94.3, p < 1e-24; TR vs. non-RR remapping: Z = 26.2, p < 1e-24. Source data

We focused on the switch days to analyze single-cell remapping patterns. First, we identified place cells as cells with significant spatial information (SI) either before or after the reward switch (means ± s.d. across 11 mice, seven switch sessions: 459 ± 263 place cells out of 954 ± 453 cells imaged; 48.5 ± 14.5% identified as place cells). We then assessed changes in the peak spatial firing of these cells before versus after the reward switch. After a switch within an environment, a subset of place cells maintained a stable field at the same track location (‘track-relative’ (TR) cells, 21.4 ± 7.9% of place cells, mean ± s.d. across six switches within environment, 11 mice; Fig. 2a and Extended Data Fig. 2a). However, many place cells remapped when the reward moved, despite the constant visual stimuli. We observed cells with place fields that disappeared (‘disappearing’; 11.2 ± 7.0%), appeared (‘appearing’; 6.8 ± 4.0%) or precisely followed the reward location similar to previous reports^22^ (‘remap near reward’; 4.8 ± 3.3%), firing within ±50 cm of the beginning of both reward zones. Notably, a subset of place cells with fields distant from reward (>50 cm) also remapped after the reward switch (‘remap far from reward’; 15.6 ± 3.6%; Fig. 2a and Extended Data Fig. 2a–d). At the population level, a reward switch within a constant environment induced more remapping than a fixed reward and environment^28,29^ but less remapping than the introduction of a novel environment^4–6^ with the reward switch (Extended Data Fig. 2b–d).

### Reward-relative remapping both near and far from reward

To visualize whether a subpopulation of cells shifted their place fields to match the shift in reward location, we circularly shifted the cells’ spatial activity on trials after the switch to align the reward locations. A subset of cells indeed remapped to a similar relative distance from reward, within and across environments (putative ‘reward-relative’ (RR) cells; see below and Methods) (Fig. 2b). This alignment relative to reward occurred for fields far from the reward zone start (>50 cm) and even for fields that, from the perspective of the linear track position, remapped from the latter half of the track to the beginning (or vice versa) despite the variable length of the teleport zone between trials (for example, Fig. 2b, cells m4.109 and m14.482). The distance run in the teleport zone did not predict spatial firing variability on the following lap in the vast majority (~93%) of RR cells (Extended Data Fig. 2e,f), suggesting that these cells do not simply track distance run from the last reward. At the same time, other cells seemed to remap randomly after the reward switch, as circular shifting did not align their fields (Fig. 2b).

We next developed a method to assess whether RR remapping occurred at greater than chance levels. First, plotting each cell’s peak spatial firing pre-switch versus post-switch revealed the TR place cells along the diagonal, as they maintained their spatial firing positions, as well as an off-diagonal band of cells at the intersection of the reward locations, which extended far beyond the immediate reward zone (Fig. 2c, far left; Extended Data Fig. 2b, middle). To align the reward zones at zero and isolate the band of cells at this intersection, we transformed the linear track coordinates to the phase of a periodic variable (that is, 0 to 450 cm becomes −π to π radians) (Fig. 2c, middle-left). Cells that fall along the unity line in this analysis putatively remap to the same relative distance from the reward. We then excluded the TR cells to focus on the remapping cells (Fig. 2c, middle-right). We calculated the difference between each cell’s peak firing position relative to the start of the reward zone pre-switch versus post-switch (a difference in relative peaks close to zero indicates RR remapping) and compared the distribution of differences to a ‘random-remapping’ shuffle (Fig. 2c, far right). We found that the fraction of place cells that exhibited RR remapping across animals (Fig. 2d) exceeded chance on the first switch day (Fig. 2e, top) and significantly increased across task experience (Fig. 2f–h and Extended Data Fig. 2g,h). The mean of the distribution of RR firing positions was significantly greater than zero (Fig. 2e, bottom), indicating that more RR place fields are in locations following reward delivery (see peak licking at the reward zone start; Fig. 1f,g and Extended Data Fig. 1a–c) than in locations preceding reward. This post-reward shift of the distribution of cells was consistent across days (Fig. 2g, bottom).

Next, to investigate the contribution of cells near the reward to above-chance levels of RR remapping, we excluded cells with peaks within iteratively larger distances on either side of the start of both reward zones. With these exclusions, we observed that above-chance RR remapping spans nearly the entire track length by the last switch day (Fig. 2i and see Extended Data Fig. 2i–l for an example exclusion within ±50 cm, a 100 cm span, greater than the ±25 cm threshold used to define reward-specific firing in previous work^22^). Furthermore, more RR firing fields were located at positions following than preceding the reward zone at distances up to ±80 cm (Extended Data Fig. 2m). However, there was no significant increase in remapping at these distances across days (Extended Data Fig. 2n), suggesting that there is more growth in the population of RR cells closer to reward. Nevertheless, RR remapping extends beyond neurons with close proximity to reward.

We implemented additional criteria (Methods and Extended Data Fig. 2o–q) to identify a robust subpopulation of RR cells for further analysis (mean ± s.d., 16.3 ± 5.3% of place cells averaged over all switch days; 13.7 ± 3.9% on the first switch, 19.4 ± 5.2% on the last switch). We refer to cells that remapped independently of RR position as ‘non-RR remapping’ (11.9 ± 3.0% of all place cells). A remaining 31.4 ± 14.3% of place cells did not show sufficiently stereotyped remapping patterns to be classified in any of the categories described here.

**Fig. 3:**
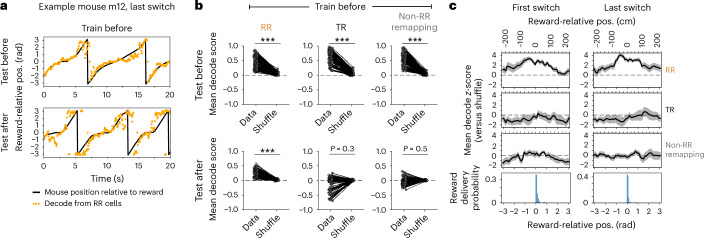
RR population provides accurate decoding of RR position. **a**, Example decode of animal’s RR position from activity of RR cells. Decoder is trained on trials before the switch and tested on held-out trials before the switch (top) or tested on trials after the switch (bottom). **b**, Mean decode score compared to the mean of shuffles per session (*n* = 77 sessions, 11 mice, seven switch days), for each subpopulation of cells classified by remapping type. ***P = 7.54 × 10^−14^, two-sided Wilcoxon signed-rank tests, Bonferroni-corrected for multiple comparisons. Effect sizes: test before: RR, 2.6; TR, 3.3; non-RR, 3.2; test after: RR, 2.7; TR, −0.5; non-RR, −0.2. **c**, Top three rows: mean ± s.e.m. decode score from each subpopulation (across *n* = 11 mice) binned as a function of RR position, after *z*-scoring to each session’s shuffle, trained on trials before and tested on trials after the switch. Bottom row: distribution of reward delivery positions on the first (left) or last (right) switch day. Note that decoding accuracy from RR cells extends far beyond the reward location. Source data

Consistent with the categorization of cells as RR, we found that we could accurately decode the animals’ RR position from the activity timeseries of RR neurons (Fig. 3a). Using a decoder trained and tested on trials before the reward switch, we could, as expected, decode RR position from either RR, TR or non-RR remapping subpopulations compared to shuffled datasets (Fig. 3b, top). However, when we tested on trials after the reward switch, only the RR population decoded the animals’ RR position better than shuffle (Fig. 3b, bottom), consistent with the preservation of RR coding both pre-switch and post-switch. Moreover, this above-shuffle decode of RR position using RR cells was significant across the majority of the environment (Fig. 3c; range of *z*-scored decode >2: −104.5 ± 20.1 cm to +152.7 ± 22.9 cm relative to reward zone start; mean ± s.e.m. across switch days).

### Independent remapping of individual place fields per cell

**Extended Data Fig. 3:**
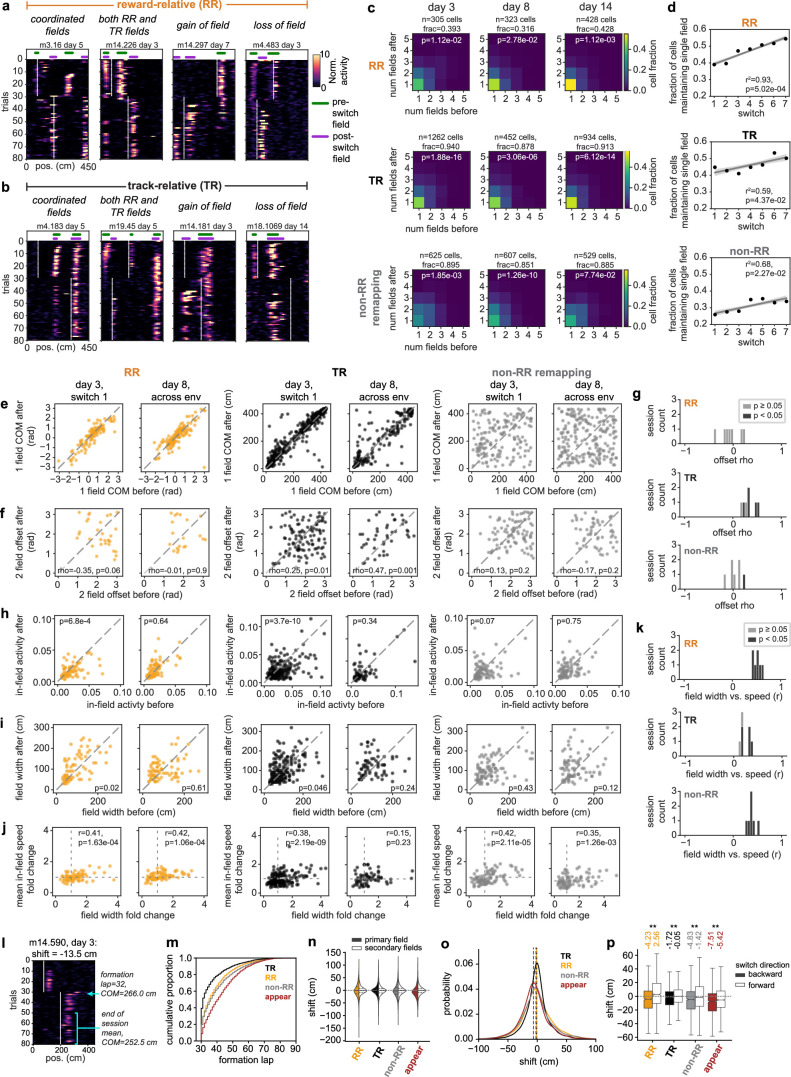
Quantification of individual place field properties. **a**) Example cells with multiple place fields, identified as reward-relative (RR) based on their peak spatial activity. Left: coordinated remapping between two RR fields; Middle-left: independent remapping, where one field is RR and another is track-relative (TR); Middle-right: gain of place field after the reward switch; Right: loss of place field after the switch. White vertical lines, reward zone starts. Green and purple horizontal lines, position bins included in each field before (green) vs. after the reward switch (purple). **b**) Example cells with multiple place fields, identified as TR based on their peak spatial activity, as in (**a**). **c**) Histograms of the number of place fields per cell before vs. after the switch, per subpopulation and day. Cell n and the fraction of total cells in each day and category captured by the place field criteria (at least 1 significant place field both before and after the switch; see Methods) is displayed above each plot; n = 11 mice. P-values from one-sided Wilcoxon signed-rank tests for more fields after vs. before the switch. Significance level with Bonferroni correction, p < 0.0024 (21 comparisons, 7 switch days and 3 subpopulations). We found no significant differences between subpopulations in field number change (post- minus pre-switch) except for days 7, 8, and 14 (Kruskal-Wallis tests, day 7: p = 3.31e-5, day 8: p = 1.99e-2, day 14: p = 6.88e-3), where post-hoc rank-sum tests revealed that these differences were explained by reward-relative cells generally having a smaller increase in the number of fields post vs. pre compared to TR and non-RR remapping cells (day 7: RR vs. non-RR-remapping Z = −3.60, p = 3.22e-4, RR vs. TR Z = −4.08, p = 4.43e-5; day 8: RR vs. non-RR-remapping Z = −2.54, p = 1.12e-2; day 14: TR vs. non-RR-remapping Z = 2.72, p = 6.56e-3). **d**) The fraction of cells maintaining a single field both before and after the reward switch increases across switch days for all subpopulations, most significantly for the reward-relative cells. Gray line and shading, best fit ± SD of linear regression. **e**) Field centers of mass (COM) for cells with single place fields before vs. after the switch, shown in radians relative to reward for RR cells and in position along the track (cm) for TR and non-RR cells. In (**e**–**i**), dashed line marks the unity line. RR: day 3 n = 119 cells, 11 mice; day 8 n = 156 cells, 11 mice; TR: day 3 n = 565 cells, 11 mice; day 8 n = 202 cells, 11 mice; non-RR: day 3 n = 162 cells, 11 mice, day 8 n = 212 cells, 11 mice. **f**) Offset (in radians) between field COMs for cells with exactly 2 fields both before and after the reward switch. Circular-circular correlation coefficients and two-sided p-values are displayed in each plot (no corrections for multiple comparisons). Note a lack of strongly coordinated remapping between pairs of fields, especially for RR cells. RR: day 3 n = 31 cells, 9 mice, day 8 n = 20 cells, 9 mice; TR: day 3 n = 118 cells, 10 mice, day 8 n = 43 cells, 9 mice; non-RR: day 3 n = 92 cells, 9 mice, day 8 n = 56 cells, 10 mice. **g**) Stacked histograms of correlation coefficients shown in (**f**) but for all sessions (n = 7 days), per subpopulation (cells pooled across mice), colored by p-value. **h**) Mean in-field deconvolved activity before vs. after the switch for cells with a single place field both before and after (see Methods). RR: day 3 n = 78 cells, 11 mice, day 8 n = 81 cells, 11 mice; TR: day 3 n = 235 cells, 11 mice, day 8 n = 66 cells, 9 mice; non-RR: day 3 n = 94 cells, 10 mice, day 8 n = 82 cells, 11 mice. Significant changes (∆) in activity after minus before the switch, at p < 0.007 (Bonferroni-corrected two-sided Wilcoxon signed-rank tests) were detected on 2 switch days for RR cells (median ∆ = −0.003), 6 switch days for TR cells (median ∆ = −0.005), and 2 switch days for non-RR cells (median ∆ = −0.005). **i**) Field width before vs. after the switch for cells with a single place field both before and after. Same n as in (**h**). Significant changes (∆) in field width after minus before the switch, at p < 0.007 (Bonferroni-corrected two-sided Wilcoxon signed-rank tests) were detected on zero days for RR cells, 2 switch days for TR cells (median ∆ = +10 cm), and zero days for non-RR cells. **j**) Fold change in field width, after vs. before the switch (positive fold change indicates a wider field), is correlated with the fold change in the animal’s mean running speed through the field. Two-sided Pearson correlation coefficient and p-value are shown; same n of cells as in (**h**). Dotted lines, fold change of 1. **k**) Stacked histograms of correlation coefficients shown in (**j**) but for all sessions (n = 7 days) per subpopulation, colored by p-value. **l**) Example identification of a reward-relative field’s formation lap (blue arrow) following the reward switch and the center of mass (COM) of firing on that lap. Blue bracket indicates COM from the mean of the last 30 trials. White lines, reward zone starts. **m**) Cumulative distributions of formation laps for the primary field (exhibiting peak activity) of each subpopulation, pooled across animals and switch days. Two-sided Wilcoxon rank-sum tests, Bonferroni corrected significance level of p < 0.0083: RR vs. TR: Z = 17.8, p = 6.5e-71; RR vs. non-RR: Z = −2.9, p = 3.4e-3; RR vs. appear: Z = −14.5, p = 2.2e-47; TR vs. non-RR: Z = −22.8, p = 1.9e-115; TR vs. appear: Z = −32.8, p = 2.1e-236; non-RR vs. appear: Z = −12.3, p = 1.5e-34 (RR n = 2368 fields, TR n = 6255 fields, non-RR n = 3445 fields, appear n = 2386 fields). **n**) Primary fields of each subpopulation show more backward and forward shifting after the formation lap than additional secondary fields. Kolmogorov-Smirnov tests between primary and secondary fields: RR: D = 0.12, p = 1.3e-7; TR: D = 0.05, p = 0.0007; non-RR: D = 0.11, p = 1.1e-11; appear: D = 0.17, p = 2.2e-15. **o**) Primary field shifts per subpopulation. Dashed lines, median shift per subpopulation. RR and non-RR cells showing more backward and forward shifts than TR cells (Kolmogorov-Smirnov tests): RR vs. TR: D = 0.08, p = 4.7e-11; RR vs. non-RR: D = 0.05, p = 6.8e-4; RR vs. appear: D = 0.12, p = 2.6e-16; TR vs. non-RR: D = 0.10, p = 2.8e-15; TR vs. appear: D = 0.17, p = 2.8e-43; non-RR vs. appear: D = 0.09, p = 1.9e-9. Appearing cells show more backward shifts than the other subpopulations (two-sided Wilcoxon rank-sum tests): RR vs. TR: Z = 0.57, p = 0.57; RR vs. non-RR: Z = 3.7, p = 2.2e-4; RR vs. appear: Z = 8.8, p = 1.2e-18; TR vs. non-RR: Z = 4.6, p = 5.2e-6; TR vs. appear: Z = 11.6, p = 2.7e-31; non-RR vs. appear: Z = 5.8, p = 8.0e-9; same n of primary fields as in (**m**). Bonferroni-corrected significance level of p < 0.004 for all tests. **p**) Primary fields exhibit more backward shifts when the reward is moved backward on the track compared to forward. Boxes, interquartile range; whiskers, 2.5th to 97.5th percentile; horizontal lines, median; notches, median confidence interval from 10,000 bootstraps. Filled boxes, sessions in which reward was switched backward on the track; open boxes, forward switches. **p < 0.01, two-sided permutation test on the two-sided Wilcoxon rank-sum Z-statistic between backward and forward switches within each subpopulation (backward and forward session labels were permuted 100 times). Median shift of fields per switch direction is shown above each boxplot. Original two-sided Wilcoxon rank-sum test results, backward vs. forward, with n of fields in each set of sessions: RR: Z = −8.9, p = 5.5e-19 (n = 1119 backward, 1249 forward); TR: Z = −6.6, p = 4.9e-11 (n = 2885 backward, 3370 forward); non-RR: Z = −5.9, p = 3.9e-9 (n = 1485 backward, 1960 forward); appear: Z = −4.1, p = 3.4e-5 (n = 965 backward, 1421 forward). Source data

Many CA1 place cells express multiple fields, and field number, firing rate and size can change with remapping^4,5,21^. In the RR, TR and non-RR subpopulations, we observed varied remapping patterns of individual place fields following reward switches (Extended Data Fig. 3), including coordinated (for example, Fig. 2b cell m4.109 and Extended Data Fig. 3a cell m3.16) and independent remapping between fields, as well as the gain or loss of fields (Extended Data Fig. 3a,b). The RR, TR and non-RR subpopulations all tended to transiently exhibit more fields after the switch (Extended Data Fig. 3c). However, the fraction of cells maintaining a single place field increased with task experience (Extended Data Fig. 3d), suggesting that the gain of place fields following a novel reward change^21^ may reflect transient plasticity that stabilizes with familiarity.

To determine whether multiple fields of the same RR cell remapped coherently or independently, we analyzed cells with two fields both before and after the switch. We found no correlation in the offset between the two fields at the population level for RR cells, indicating largely independent remapping of fields (Extended Data Fig. 3f,g). Therefore, although our remapping categorization system captures the dominant mode of each cell’s remapping based on the field with the highest activity, RR and TR properties can be expressed at the level of individual fields.

Finally, across the RR, TR and non-RR subpopulations, we observed modest reductions of in-field deconvolved activity (that is, firing rate) (Extended Data Fig. 3h) and, seldomly, a significant bias in place field width change, which may be driven by changes in running speed^30,31^ (Extended Data Fig. 3i–k). In addition, some cells showed backwards field shifts after the formation trial (Extended Data Fig. 3l–p), characteristic of behavioral timescale synaptic plasticity^30,31^, while other cells showed forward shifts. At the population level, appearing cells showed more backwards shifting (Extended Data Fig. 3o) and later formation laps than the other subpopulations (Extended Data Fig. 3m). Notably, the degree of backward and forward shifting depended on whether the reward moved backward or forward on the track, respectively (Extended Data Fig. 3p). Together, these results highlight the heterogeneous manner in which individual place fields re-organize in response to reward location changes.

### Preserved behavioral timescale sequences relative to reward

**Fig. 4:**
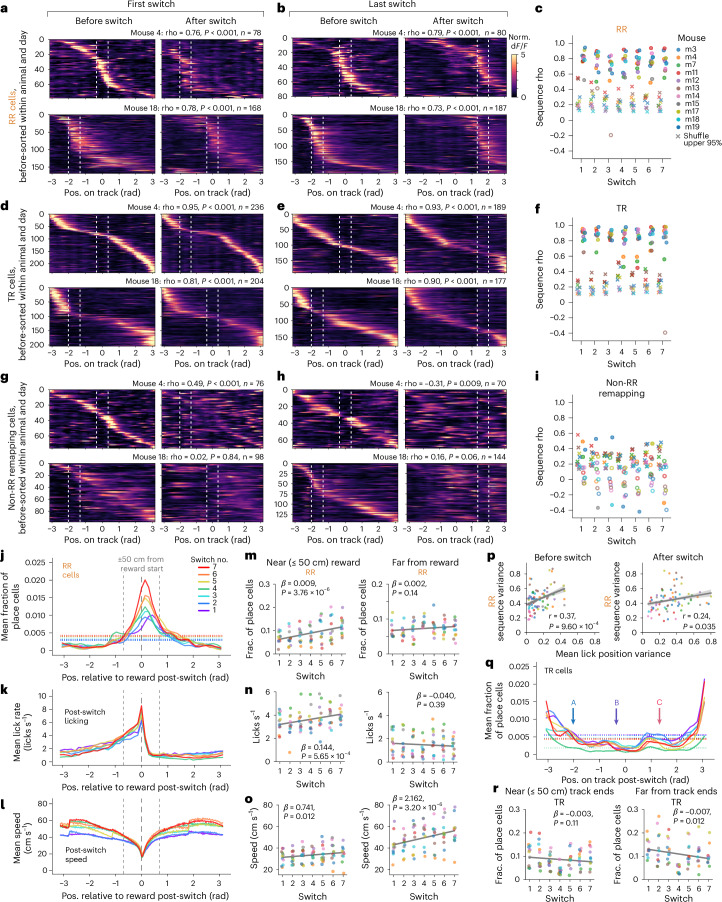
Behavioral timescale sequences relative to reward and space. **a**,**b**, Example RR cells, sorted by cross-validated peak activity before the switch on the first (**a**) or last (**b**) switch day. White dashed lines, reward zone boundaries. Sequence circular–circular correlation coefficient (rho), *P* value relative to shuffle (two-sided permutation test in **a**–**i**), number of cells shown above. Color scale applied in **d**, **e**, **g** and **h**. **c**, Correlation coefficients for RR sequences (*n* = 11 mice). ‘X’, upper 95th percentile of 1,000 cell ID shuffles. Closed circles, *P* < 0.05; open circles, *P* ≥ 0.05. Mouse color key applied to **f**, **i**, **m**–**p** and **r**. **d**–**f**, TR sequences on the first (**d**) and last (**e**) switch day for mice in **a** and **b**, and correlation coefficients as in **c** (**f**). **g**–**i**, Sorted non-RR remapping cells (**g**,**h**), same mice as above, and correlation coefficients as in **c** (**i**). **j**, Distribution of RR cell peaks, post-switch on each day (fraction of place cells per animal, averaged across mice; *n* = 11 mice in **j**–**l** and **q**; s.e.m. omitted for visualization). Horizontal dotted lines, expected uniform distribution per day; vertical gray dashed lines, reward zone start ±50 cm. **k**,**l**, Mean post-switch lick rate (10 cm bins) (**k**) and running speed (2 cm bins) (**l**) colored as in **j**. Line discontinuities reflect the teleport period. **m**–**o**, Changes in RR sequence density (fraction (frac.) of place cells per mouse; **m**), mean lick rate (**n**) and mean speed (**o**) across days, ≤50 cm (left) or >50 cm (right) from the reward zone start. In **m**–**o** and **r**, *β* coefficients and *P* values (two-sided Wald test) are from linear mixed-effects models with switch index as a fixed effect and mice as random effects. **p**, Circular variance in licking positions significantly correlates with RR sequence variance before (left) and after the switch (right). Each dot is a session (*n* = 77 sessions, 11 mice). Gray line and shading, best fit ± s.d. of linear regression. **q**, Distribution of TR cell peaks, post-switch on each day as in **j**; track coordinates converted to radians (not reward-relative). Arrows indicate reward zone starts (‘A’, ‘B’, ‘C’). Horizontal dotted lines, expected uniform distribution per day. **r**, Changes in TR sequence density as in **m**, within ≤50 cm from either end of the track (left) or >50 cm from the track ends (right). Source data

**Extended Data Fig. 4:**
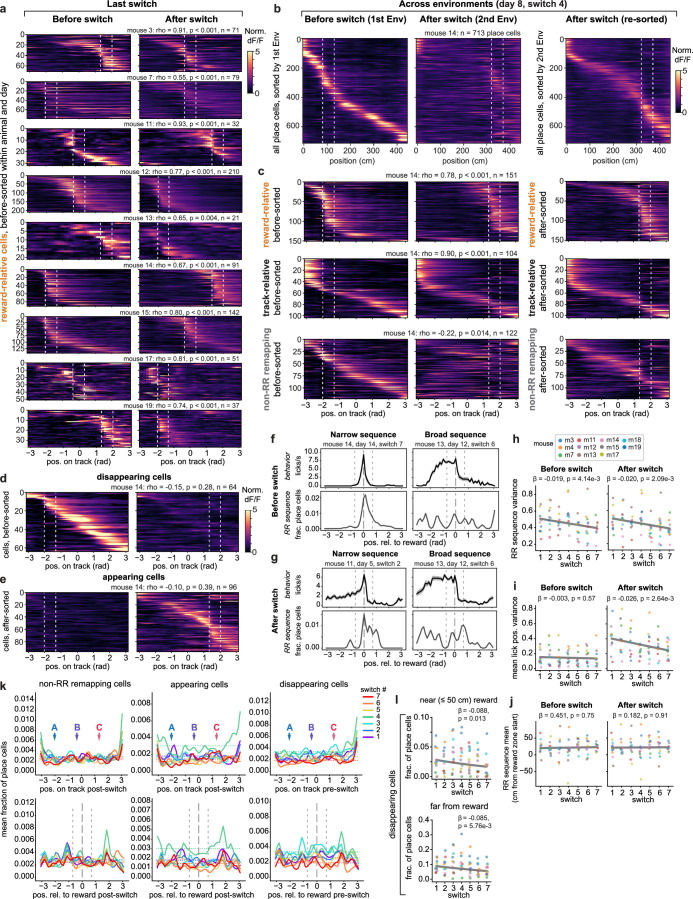
Behavioral timescale sequences across animals, remapping types, and environments. **a**) Reward-relative sequences on the last switch (day 14) for all mice not shown in Fig. 4a. In (**a**, **c**–**e**), circular-circular correlation coefficient (rho) of sequence order, p-value from two-sided permutation test, and n of cells are shown for each mouse. **b**) Population-level remapping from all place cells in example mouse m14 upon the novel environment switch (day 8). In (**b**, **c**), left two panels: cross-validated sort by firing order in Env 1; right-hand panel: cross-validated sort by firing order in Env 2. **c**) Remapping (or lack thereof) of specific subpopulations in mouse m14 upon the novel environment switch (day 8). Top: reward-relative (RR) sequences are preserved across environments; Middle: a subset of track-relative place cells retain their firing order across environments, especially at the beginning of the track; Bottom: non-RR remapping cells remap globally, mirroring the overall population. **d**, **e**) Example sequences of disappearing cells (**d**) and appearing cells (**e**) from mouse m14 on the last switch (day 14). In (**d**, **e**), cross-validated sort was performed on the trials in which cells had significant spatial information (before for disappearing, after for appearing). Left: before switch; Right: after switch. **f**) Example mean ± SEM licking (top) and distribution of RR sequence peak firing positions (bottom) during trials before the switch, aligned to the start of the reward zone. Left: example narrow RR sequence (low circular variance around reward); Right: example broad sequence (high circular variance). Note more precise licking coinciding with a sequence that is more dense around the reward zone (left). Vertical gray dashed lines, reward zone start and surrounding ±50 cm. **g**) Same as (**f**), but showing example licking and RR sequences for trials after the switch. **h**) RR sequence variance decreases across experience. In (**h**–**j**, **l**), each dot is a mouse (n = 11 mice), and regression coefficients β and p-values (two-sided Wald test) are from linear mixed effects models with switch index as the fixed effect and mice as random effects. **i**) Mean licking position variance after the switch decreases across switch days. **j**) The mean reward-relative position of RR cell sequences is static across days. **k**) Distributions of peak firing positions on each switch day (fraction of place cells per animal, averaged across n = 11 mice, SEM omitted for clarity) for non-RR remapping cells (left), appearing cells (middle), and disappearing cells (right). Top row: by linear track position, converted to radians. Arrows, start of each reward zone. Bottom row: by position relative to the reward zone start. Horizontal dotted lines, expected uniform distribution for each day. Vertical gray dashed lines, reward zone start and surrounding ±50 cm. **l**) Both near ( ≤ 50 cm, top) and far from ( > 50 cm, bottom) the start of the reward zone, the fraction of place cells with disappearing fields decreases across experience. Non-RR remapping and appearing fractions did not show significant changes over experience. Source data

**Extended Data Fig. 5:**
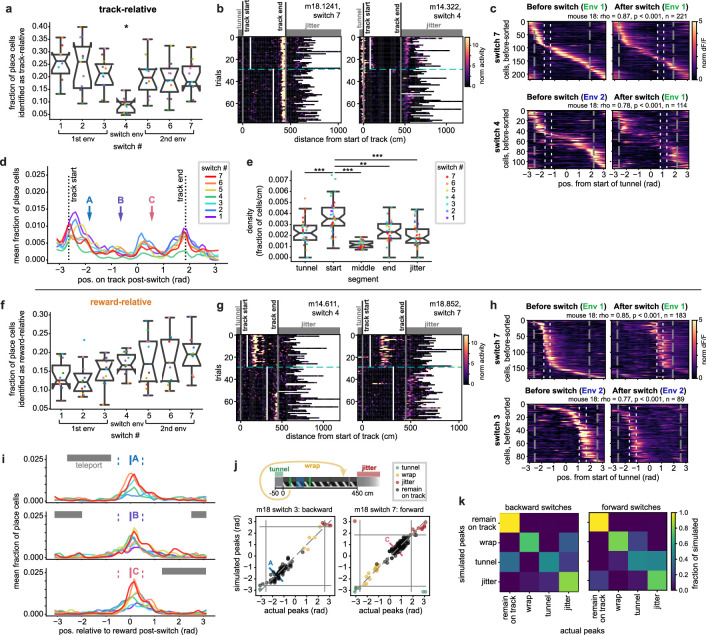
Track-relative and reward-relative activity during the teleport period. **a**) Fraction of place cells identified as track-relative on each switch day. Colored dots, fractions for each mouse (n = 11 mice, see Fig. 4 for color key). *p < 0.05, significant effect of switch day, post-hoc two-sided Wilcoxon signed-rank tests, Holm-corrected for multiple comparisons, following Friedman test (day Q = 32.4, p = 1.4e-5, degrees of freedom = 6): switch 1 vs. 4 p = 0.02, switch 2 vs. 4 p = 0.02, switch 3 vs. 4 p = p = 0.02, switch 5 vs. 4 p = 0.02, switch 6 vs. 4 p = 0.031, switch 7 vs. 4 p = 0.02 (all other comparisons n.s.). In (**a**, **e**, **f**): boxes, interquartile range; whiskers, 2.5th to 97.5th percentile; horizontal lines, median; notches, median confidence interval from 10,000 bootstraps. **b**) Activity of two example track-relative cells as a function of distance run from the start of the track, including the variable distance during the “jitter” period of the teleport (unchanging gray display for a randomized time interval). Gray bars mark the whole teleport period, including the “tunnel” (during which the gray display moved) at distance −50 to 0 cm preceding the start of the track. White regions are unoccupied distance bins. Left: example with firing bound to the track; Right: example with firing in the tunnel and jitter. In (**b**, **c**, **g**, **h**): gray lines, ends of the track. In (**b**, **g**): white lines, reward zone starts; horizontal teal line, reward zone switch trial. **c**) Example behavioral timescale sequential firing of track-relative place cells for two switches, including cells that fire during the teleport period, in coordinates from the start of the tunnel, converted to radians. In (**c**, **h**): white dotted lines, reward zone boundaries. Note higher sparsity of activity on the track when the environment changes (bottom) compared to when the environment is fixed (top) (note also “Env 2” is the first environment experienced for this animal, m18). **d**) Distribution of track-relative peak firing positions, post-switch on each switch day (mean across n = 4–7 mice per day), in coordinates from the start of the tunnel, converted to radians, binned to the maximum distance reached with fewer than 10 missing trials per bin in a session (580 cm). Arrows indicate the start position of each reward zone (“A”, “B”, “C”). Expected mean uniform distributions (see Fig. 4) are omitted since different animals and days have different occupancy of the bins following the track end. 32.7 ± 10.8% (mean ± SD, n = 7 mice with any teleport sampling) of track-relative cells peaked within either the jitter or tunnel on days in which the environment was constant, 41.9 ± 5.4% on the day in which the environment changed (n.s. effect of day: Q = 11.0, p = 0.087, degrees of freedom = 6, Friedman test, n = 4 mice sampled on all days). **e**) Density of track-relative cell firing peaks per task segment (density = fraction of track-relative cells divided by spatial distance of segment). Tunnel: −50 to 0 cm; start: 0 to 50 cm; middle: 50 to 400 cm; end: 400 to 450 cm; jitter: 450 to 580 cm. **p < 0.01, ***p < 0.001, coefficient t-tests after Benjamini-Yekutieli correction for multiple comparisons, linear mixed effects model with fixed effects of segment (referenced to start of track), switch day, interaction of segment and switch day, and mice as random effects. Significant fixed effects with respect to start segment: tunnel minus (–) start (β = −0.0026, p = 1.2e-7), middle – start (β = −0.0029, p = 2.5e-9), end – start (β = −0.0016, p = 1.6e-3), jitter – start (β = −0.0018, p = 7.2e-4), switch day x [tunnel – start] (β = 0.0004, p = 5.5e-3). **f**) Fraction of place cells identified as reward-relative on each switch day. Colored dots, fraction for each mouse, as in (**a**) (n = 11 mice). **g**) Activity of two example reward-relative cells as a function of distance run from the start of the track, as in (**b**). Left: example with firing bound to the track; Right: example where the cell’s field remapped into the jitter. **h**) Example behavioral timescale sequential firing of reward-relative place cells for two switches, including activity during the teleport period, as in (**c**). **i**) Distributions of reward-relative peak firing positions, post-switch on each switch day, with sessions split by reward zone location “A” (top), “B” (middle), or “C” (bottom; mean across n = 1–4 mice with reward zones at each location per day), colored as in (**d**) but aligned to the reward zone start (dotted lines, ±50 cm). Gray bars, bins included in the teleport period. 12.7 ± 7.1% of reward-relative cells (mean ± SD, n = 7 mice with any teleport sampling) had peak firing within the teleport period (tunnel or jitter) before or after the switch on days with a constant environment; 27.2 ± 5.7% on the day when the environment changed (effect of day: Q = 11.1, p = 0.084, degrees of freedom = 6, Friedman test, n = 4 mice sampled on all days). **j**) Top: schematic of virtual track and teleport zones. Bottom: Example comparisons of actual remapping position vs. simulated position post-switch if reward-relative cells remapped the exact distance the reward zone moved backward (bottom left) or forward (bottom right), corresponding to the example sessions shown in (**h**). Each point is a cell, colored by its simulated remapping destination. “Wrap”: the cell’s field should move sufficiently to wrap around the track to the opposite side of the reward zone from where it started. “Remain on track”: cells that remain within the track boundaries when they remap. “Jitter”: fields that should remap into the jitter period. “Tunnel”: fields that should remap into the tunnel. Solid gray lines, track starts and ends; dashed gray line, unity line; colored hashes, starts of reward zones. **k**) Summary of actual vs. simulated reward-relative remapping for backward (left) and forward (right) switches across n = 4–7 mice, 7 switch days. Each bin contains the fraction of simulated cell positions that exhibited the actual remapping position of the columns (each row is normalized to its sum). Source data

Next, we asked whether the RR cells constructed behavioral timescale sequences of activity anchored to reward, defined by preservation of firing order between cells regardless of reward location. We first sorted the RR cells within each animal by their peak firing position on trials before the reward switch, using split-halves cross-validation (Methods). This sorting was applied to the post-switch trials, revealing a strong preservation of firing order relative to reward within and across environments (Fig. 4a,b and Extended Data Fig. 4a–c), despite global remapping in the overall place cell population when we introduced the novel environment (Extended Data Fig. 4b,c). Sequence preservation (the circular–circular correlation coefficient between peak firing positions pre-switch versus post-switch) was higher than the shuffle of cell identities for nearly all animals and days (75 out of 77 sessions) (Fig. 4c). These sequences spanned the task structure from reward to reward, with cells firing the furthest from reward occasionally ‘wrapping around’ from the beginning to the end of the track or vice versa (Fig. 4a,b and Extended Data Fig. 4a). The TR place cells likewise constructed robust behavioral timescale sequences (76 out of 77 sessions) relative to the ends of the track, within (Fig. 4d–f) and across environments (Extended Data Fig. 4c, middle). However, the environment change significantly reduced the proportion of cells identified as TR (Extended Data Fig. 5a; 8.4 ± 3.1% of place cells across environments on day 8 versus 21.4 ± 7.9% within; mean ± s.d., *n* = 11 mice), indicating that a subset of TR cells may encode the similar structure between environments while the majority are influenced by environment identity. In contrast to the RR and TR subpopulations, sequential firing was not preserved for most of the non-RR remapping cells (61 out of 77 sessions did not exceed shuffle; Fig. 4g–i and Extended Data Fig. 4c, bottom) or for disappearing and appearing cells (Extended Data Fig. 4d,e).

### RR representation increased with learning

We next quantified the distribution of peak spatial firing for each subpopulation relative to either reward or the track (Fig. 4j–r). RR sequences spanned the entire track but over-represented a region from ~50 cm before to ~70 cm after the reward zone start, with the highest density of cells following the start of the reward zone (Fig. 4j). This density near reward increased with task experience, while the fraction of place cells >50 cm from the reward zone start remained stable over days (Fig. 4j,m). In parallel, mice increased their post-switch anticipatory lick rate (Fig. 4k,n) and running speed over days (Fig. 4l,o). These changes suggest that the number of neurons allocated to the RR sequences within the overall population increases as behavioral performance improves. Additionally, on any given day, the precision of the animal’s licking correlated with the precision of the RR sequence (that is, the width of the peak activity distribution around reward, measured as the circular variance) (Fig. 4p and Extended Data Fig. 4f,g). The variance of both the RR distribution and the post-switch licking decreased across switch days (Extended Data Fig. 4h,i) but the mean position of the sequence did not change (Extended Data Fig. 4j), suggesting an increasingly precise neural representation as behavior became refined. In contrast to the RR sequences, the TR sequences tended to over-represent the ends of the track rather than the reward zones (Fig. 4q). TR sequence density decreased at positions far from the track ends across days, consistent with selective stabilization of place fields near the landmarks^20,32,33^ provided by the environment boundaries (Fig. 4r). By comparison, the disappearing, appearing and non-RR remapping cells encoded a more uniform representation (Extended Data Fig. 4k,l).

To understand how the task event of the teleport influenced each subpopulation, we examined activity throughout the variable length teleport period. The density of the TR representation remained highest at the start and end of the track (Extended Data Fig. 5b–e), indicating that the edges of the virtual environment, rather than the teleport, provide the most salient anchors for the TR code. By contrast, the RR code was not bound by the virtual environment, as there was no decrease in the proportion of RR cells upon the environment switch (Extended Data Fig. 5f). The RR sequences extended sparsely into the teleport periods (Extended Data Fig. 5g–i), with a close correspondence between cells’ peak firing positions following the reward switch and where they ‘should’ be if they remapped by the exact distance that the reward zone moved, even into the teleport period (Extended Data Fig. 5j,k). These observations suggest that the RR code is anchored to the reward zone and maps the cyclical nature of the task from reward to reward.

### Dynamic cell recruitment into the RR population

**Fig. 5:**
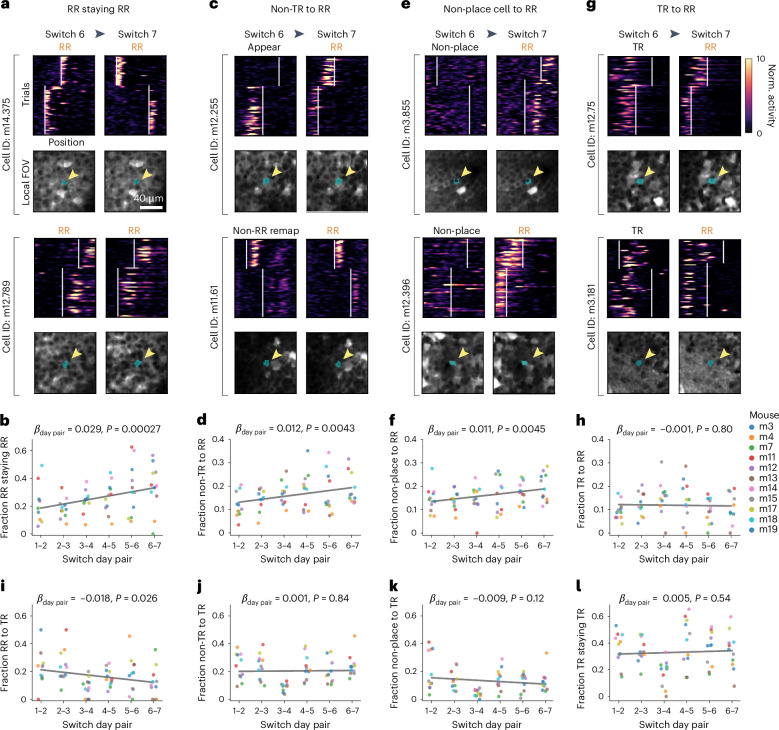
Increased recruitment into the RR population over days. **a**, Example cells followed from switch 6 to 7 (days 12–14) that maintained their RR firing pattern across days. Top of each cell: spatial activity per trial on each day, normalized to the mean of each cell. White vertical lines, reward zone starts. Bottom of each cell: mean local FOV around the cell each day (blue shading and yellow arrowhead indicate cell ROI). **b**, Fraction of followed RR cells on each switch day that remain RR on subsequent switch days increases over task experience. In **b**, **d**, **f**, **h** and **i**–**l**, all *β* coefficients and *P* values (two-sided Wald test) are from linear mixed-effects models with switch day pair as a fixed effect and mice as random effects (*n* = 11 mice in each plot; key in **h**). Gray line, model fit. **c**, Same as **a** but for two example cells that were identified as appearing (top) or non-RR remapping (bottom) on switch 6 but were converted to RR on switch 7. **d**, Fraction of ‘non-TR’ place cells (combined appearing, disappearing and non-RR remapping) that convert into RR cells on subsequent switch days increases over task experience. **e**, Same as **a** but for two example non-place cells on switch 6 (did not have significant SI in either trial set) that were converted to RR place cells on switch 7. **f**, Fraction of non-place cells converting to RR cells on subsequent switch days shows a modest increase over task experience. **g**, Same as **a** but for two example TR cells on switch 6 that were converted to RR on switch 7. **h**, Fraction of TR cells converting to RR cells does not change over task experience. **i**, Fraction of RR cells converting to TR cells decreases slightly over task experience. **j**, Fraction of non-TR cells (combined appearing, disappearing and non-RR remapping) converting to TR does not change significantly. **k**, Fraction of non-place cells converting to TR does not change significantly. **l**, Fraction of TR cells remaining TR does not change significantly. Source data

**Extended Data Fig. 6:**
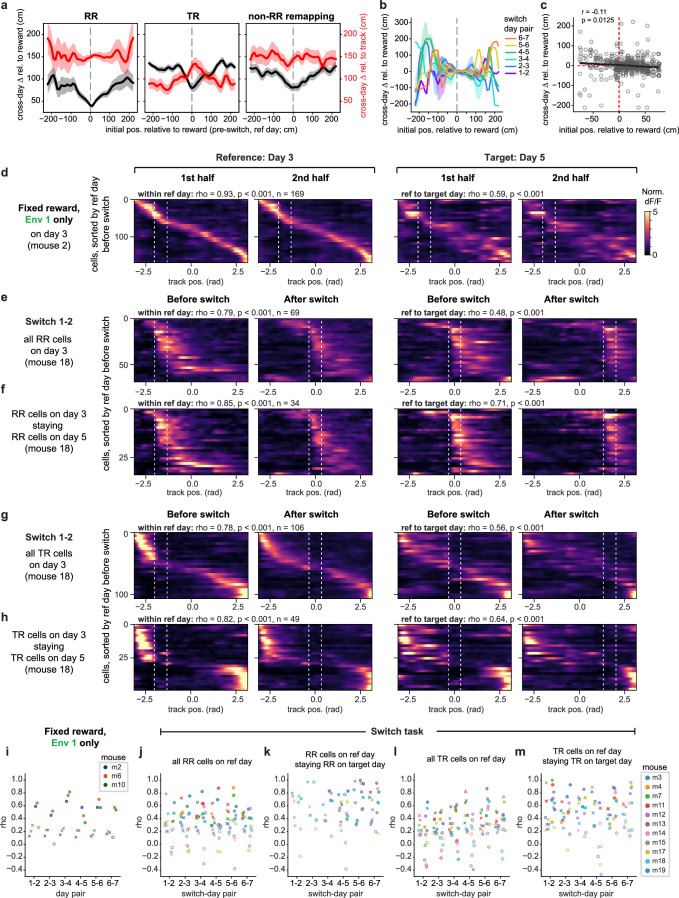
Dynamics of cells and sequences followed across days. **a**) Cross-day change (∆) in peak firing position relative to reward (black; circular distances converted to cm) or relative to the linear track (red), as a function of initial proximity to the reward zone start, for cells followed from one switch day to the next. Data are shown as mean ± SEM across pooled cells and mice, with peak firing identified from pre-reward-switch trials each day, in 10 cm bins smoothed with a 10 cm SD Gaussian. Cells are described by their remapping category on the first switch day in each pair (reference day), agnostic of category on the subsequent switch day (target day). RR n = 2294 cells; TR n = 3408 cells; non-RR remapping n = 2256 cells, 11 mice. Gray vertical line marks 0 to aid visualization. Note increased cross-day stability specifically for reward-relative firing for cells initially closer to reward. **b**) Cross-day signed change in peak spatial firing relative to reward, for reward-relative cells that stayed reward-relative across each pair of switch days, expressed as mean ± SEM across pooled cells (n = 667 cells). **c**) Initial position relative to reward on reference day is weakly anticorrelated (black line, fit of linear regression) with change in reward-relative position, for reward-relative cells (dots, n = 551 cells) that stayed reward-relative across switch day pairs (within 80 cm of the reward zone start, as this is the maximum distance contained within the track boundaries for all reward locations). That is, cells with fields preceding reward shift slightly forward toward the reward across days, and cells with fields following reward shift slightly backward toward the reward. Dashed red lines, x = 0 and y = 0 to aid visualization. **d**) Example place cell sequence followed from days 3 to 5 in an animal (m2) from the fixed-condition cohort (same reward zone and environment for all 14 days), where neurons were identified using the same criteria as reward-relative cells (requiring field position relative to reward to be stable from the 1st to the 2nd half of trials), but without a reward switch to identify reward-relative cells, this likely includes other remapping categories. In (**d**–**h**), cells within animal are sorted by their cross-validated peak activity either in the 1st half of trials or before the switch on the reference (ref) day; the sequence circular-circular correlation coefficient (rho), p-value relative to shuffle (two-sided permutation test), and n of cells followed across the day pair are shown above each plot; across-day correlation is computed between the 2nd trial set on the ref day and the 1st trial set on the target day. In (**d**), note that drift is apparent across days in this fixed-condition animal as denoted by the drop in correlation coefficient. **e**) Reward-relative cells from day 3 (switch 1), mouse m18 (same animal as in Fig. 4 examples) that were followed to day 5 (switch 2), agnostic of the remapping types for the cells on day 5. **f**) Reward-relative cells from day 3, m18 that were followed to day 5 and remained reward-relative on day 5. **g**) Track-relative cells from day 3, m18 that were followed to day 5, agnostic of the remapping types for the cells on day 5. **h**) Track-relative cells from day 3, m18 that were followed to day 5 and remained track-relative on day 5. **i**) Across-day correlation coefficients for place cell sequences followed across day pairs in the fixed-condition cohort, as described in (**d**) (n = 3 mice; 95 ± 54 followed cells per day pair, mean ± SD across mice and day pairs). In (**i**–**m**), “x” marks the upper 95^th^ percentile of 1000 shuffles of cell IDs (jittered to the left of each set of coefficients, color-coded by animal). Closed circles, p < 0.05 (two-sided permutation test); open circles, p ≥ 0.05. **j**) Across-day correlation coefficients for reward-relative sequences followed across day pairs, agnostic of the cells’ remapping types on the target day, for the “switch” task animals (n = 11 mice; 34 ± 26 cells, mean ± SD across mice and day pairs; one outlier not shown with rho < −0.4). **k**) Across-day correlation coefficients for reward-relative sequences remaining reward-relative on each target day (n = 9 mice with at least 5 followed cells on at least 1 day pair; 9 ± 11 cells, mean ± SD across mice and day pairs). Note slightly higher correlation coefficients compared to (**j**), though the small numbers of cells that were both successfully followed and remained reward-relative preclude a robust comparison. **l**) Across-day correlation coefficients for track-relative sequences followed across day pairs, agnostic of the cells’ remapping types on the target day (n = 11 mice; 51 ± 37 cells, mean ± SD across mice and day pairs). **m**) Across-day correlation coefficients for track-relative cells remaining track-relative on each target day (n = 10 mice; 17 ± 16 cells, mean ± SD across mice and day pairs). Source data

The growth in RR sequence density with task experience raised three possibilities: RR remapping becomes more consistent, more cells are recruited to the RR population or a combination of both. To investigate these possibilities, we followed the activity of the same neurons across pairs of switch days. We found that across experience, RR cells were increasingly likely to remain RR on consecutive switches (Fig. 5a,b). In addition, an increasing proportion of non-TR place cells (appearing, disappearing and non-RR remapping) and non-place cells were recruited into the RR population with experience (Fig. 5c–f). TR cells were sometimes also recruited, though this occurred at a constant rate over days (Fig. 5g,h). By contrast, the TR population did not recruit more cells from any other population (Fig. 5i–k) and did not show an increased likelihood of remaining TR across experience (Fig. 5l). Altogether, RR coding became more consistent across experience and recruited cells exhibiting high flexibility (for example, appearing or disappearing fields), without decreasing the proportion of TR cells. Furthermore, RR cells that originally fired closer to reward were more likely to have stable RR tuning over days, and TR cells closer to reward were more likely to remap with respect to the track and become RR (Extended Data Fig. 6a). RR cells near reward shifted their peak firing even closer to the reward zone start over days (Extended Data Fig. 6b,c). These single cell changes are consistent with the decrease in RR sequence variance across learning (Extended Data Fig. 4h).

The TR population showed steady turnover over days (Fig. 5l), as did the fixed-condition animals (*n* = 3 mice) (Extended Data Fig. 6d), consistent with representational drift^28,29^. Drift in RR and TR sequences was slightly higher than in fixed-condition animals (Extended Data Fig. 6e–m), reflecting increased remapping from reward switches compared to a fixed reward (Extended Data Fig. 2b–d). Although sequence preservation across switch days was greater than chance in some sessions, correlation coefficients across days were generally lower (Extended Data Fig. 6j–m) than within-day sequences (Fig. 4c,f). Thus, RR and TR ensembles exhibit similar plasticity across days of experience^28,29^.

**Extended Data Fig. 7:**
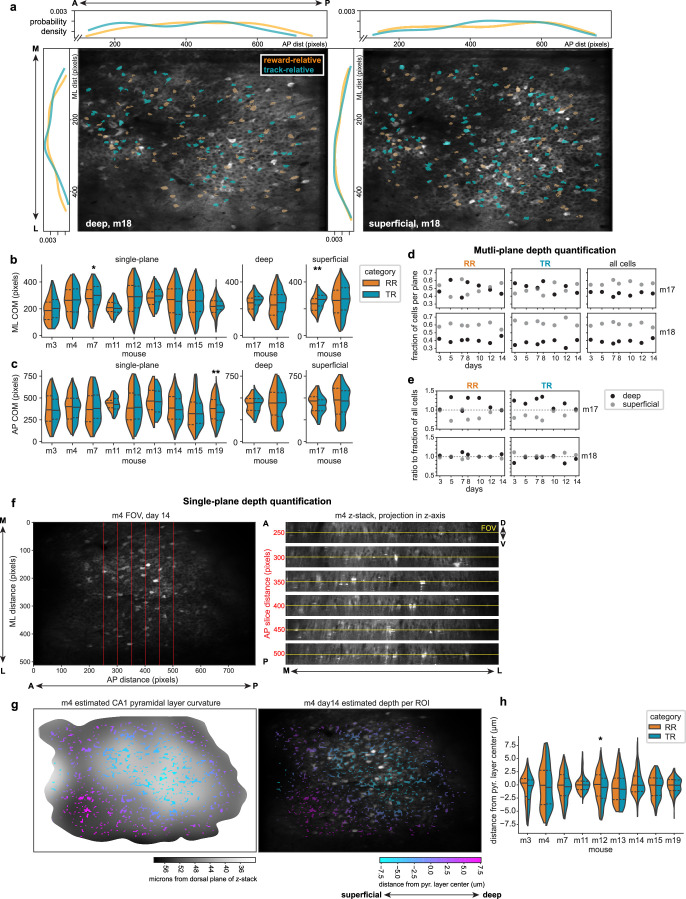
Anatomical distributions of reward-relative and track-relative cells. **a**) Example multi-plane imaging session (mouse m18, day 14) showing the mean field of view (FOV) in deep CA1 (left) and superficial CA1 (right). Cell ROIs are colored by their remapping category on this day (orange = reward-relative, blue = track-relative). Plots to the left and above each image show the kernel density estimate of the center of mass (COM) of each ROI in the ML and AP axes, respectively. Note that the FOV is shown in pixels (x = 796 pixels, y = 512 pixels) rather than microns for quantification purposes, and here the FOV is slightly truncated in the AP (x) axis due to the deadband where the laser turns around during bidirectional imaging. **b**) Distributions of the COM of each ROI in the ML axis, measured within each FOV, per mouse and remapping category. Deep and superficial planes of multi-plane animals are shown separately. Cell counts from all 7 switch days are combined as no discernible differences were observed across days. In (**b**, **c**), *p < 0.0038 Bonferroni-corrected significance level, **p < 0.0001, two-sided Wilcoxon rank-sum tests between RR and TR cells. Single-plane animals: m3: Z = 2.59, p = 9.51e-3, n = 427 RR, 440 TR cells; m4: Z = 0.27, p = 0.79, n = 366 RR, 955 TR cells; m7: Z = 3.22, p = 1.26e-3, n = 418 RR, 225 TR cells; m11: Z = 0.80, p = 0.42, n = 138 RR, 197 TR cells; m12: Z = 0.53, p = 0.59, n = 867 RR, 872 TR cells; m13: Z = 1.90, p = 0.058, n = 181 RR, 138 TR cells; m14: Z = 0.26, p = 0.79, n = 722 RR, 1077 TR cells; m15: Z = 0.01, p = 0.99, n = 967 RR, 1011 TR cells; m19: Z = 1.14, p = 0.25, n = 295 RR, 375 TR cells. Multi-plane animals: m17 deep: Z = 2.72, p = 6.57e-3, n = 137 RR, 240 TR cells; m18 deep: Z = 1.51, p = 0.13, n = 535 RR, 465 TR cells; m17 superficial: Z = 4.20, p = 2.86e-5, n = 141 RR, 237 TR cells; m18 superficial: Z = 2.47, p = 0.014, n = 785 RR, 795 TR cells. **c**) Same as (**b**) but in the AP axis. Single-plane animals: m3: Z = 0.40, p = 0.69; m4: Z = 0.28, p = 0.78; m7: Z = 1.19, p = 0.23; m11: Z = 0.95, p = 0.34; m12: Z = 0.47, p = 0.641; m13: Z = 0.41, p = 0.68; m14: Z = 2.03, p = 0.043; m15: Z = 0.40, p = 0.69; m19: Z = 3.31, p = 9.38e-4. Multi-plane animals: m17 deep: Z = 0.00, p = 0.998; m18 deep: Z = 0.03, p = 0.98; m17 superficial: Z = 2.85, p = 4.40e-3; m18 superficial: Z = 1.16, p = 0.25. **d**) Fractions of cells in each remapping category observed in the deep (black) and superficial (gray) planes of the multi-plane animals, m17 (top row) and m18 (bottom row). Right-hand columns show a bias for more cells to be detected in general in the superficial sublayer, consistent with its higher cell density. **e**) Fractions of cells in each remapping category shown in (**d**) expressed as a ratio to the fraction of all cells detected per plane, demonstrating no clear bias for RR or TR cells to either the deep or superficial sublayer. **f**) Estimation of depth per ROI in single-plane animals, shown for example mouse m4. Centered at the imaging FOV (left), a z-stack is taken from −100 to +100 microns around the FOV. Red lines illustrate mediolateral slices (right; z-stack side-views). Yellow lines mark the plane of the FOV. **g**) Estimated CA1 pyramidal layer curvature (see Methods) for example mouse m4 (left) and overlaid ROI depth estimate from day 14 (duplicated on the mean FOV, right). ROIs are color coded by estimated distance from the center of the pyramidal layer, where blue corresponds to the superficial sublayer and pink to the deep sublayer. Clipping mask (left) marks 110% of the kernel density estimate of the region containing ROIs. **h**) Distributions of the estimated depth of each ROI in the DV axis, per single-plane mouse and remapping category. Same n as in (**b**, **c**). *p < 0.0055 Bonferroni-corrected significance level, two-sided Wilcoxon rank-sum tests between RR and TR cells. m3: Z = 2.71, p = 6.74e-3; m4: Z = 0.44, p = 0.66; m7: Z = 1.53, p = 0.13; m11: Z = 0.11, p = 0.92; m12: Z = 3.08, p = 2.06e-3; m13: Z = 0.17, p = 0.86; m14: Z = 0.30, p = 0.77; m15: Z = 0.41, p = 0.68; m19: Z = 0.06, p = 0.95. Source data

Despite this flexibility, the possibility remained of an anatomical bias for either subpopulation, given previous work suggesting goal-related coding in the deep sublayer of CA1 (refs. ^34,35^). However, we found no reliable bias in the medio-lateral, antero-posterior or superficial-deep distributions of either subpopulation (Extended Data Fig. 7). Altogether, these findings highlight the flexible recruitment of RR coding at the population level rather than through dedicated cell types, with an increased allocation of neurons over learning to the RR representation.

### Encoding of reward proximity versus movement covariates

The animal’s approach to and departure from the reward zone involves stereotyped behaviors that are integral to the animal’s experience surrounding reward. To understand which features of experience are encoded in the RR representation, we took two approaches to disentangle these features and control for movement-related covariates.

**Extended Data Fig. 8:**
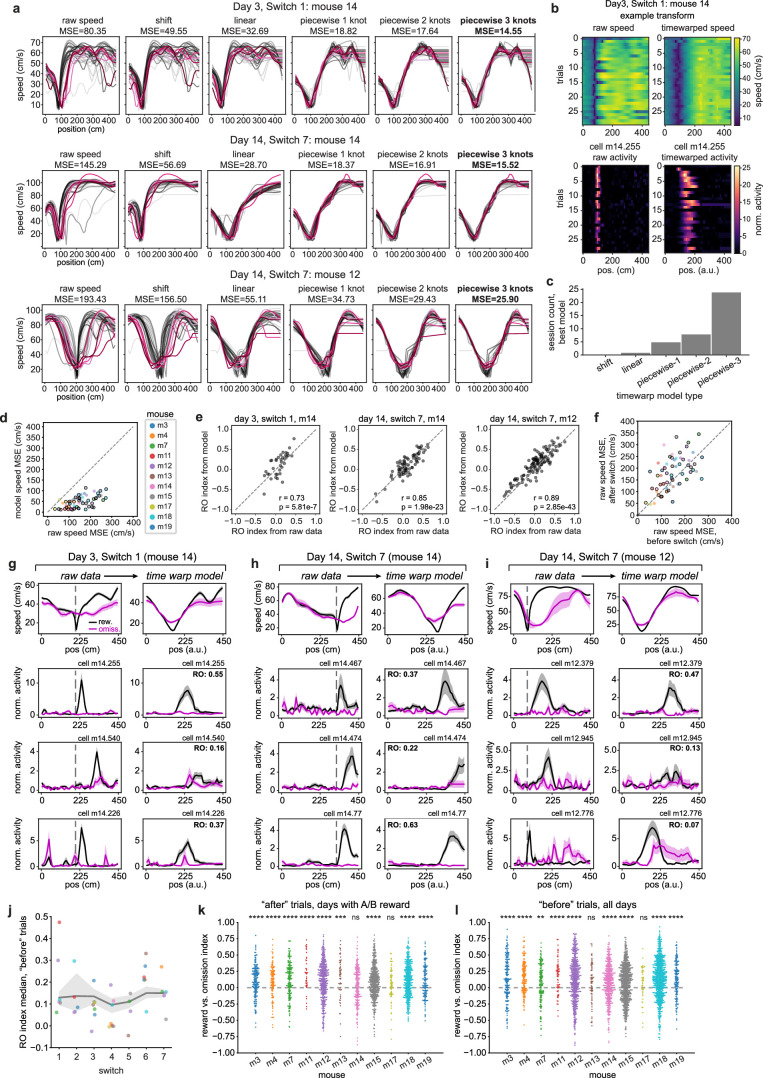
Time warp models and controls for analysis of rewarded vs. omission trials. **a**) Illustration of time warp model fitting procedure to running speed profiles. Shades of black, rewarded trials; shades of magenta, omission trials. Left to right: raw speed, followed by the transformed speed from fitting 5 model types to identify a model for best fit, as assessed by mean squared error (MSE) across transformed speed profiles per trial. Bold text, best fit. Top to bottom: the same example sessions shown in Fig. 6a–c. **b**) Example transformation using the piecewise 3 knots model fit on the speed from day 3, mouse 14 (shown in **a**, top). Top left: raw trial-by-trial speed. Top right: time warp transformed speed. Bottom left: raw, trial-by-trial binned deconvolved activity (normalized to the mean of the session) for example cell m14.255, also shown in Fig. 6a. Bottom right: time warp transformed neural activity of cell m14.255. **c**) Frequency with which each model type was selected as maximally aligning speed according to MSE, in an initial cohort of n = 7 mice. We subsequently applied piecewise-3 to all data to avoid variance in results due to model type. **d**) Time warp model fitting significantly reduces MSE across trial-by-trial speed profiles compared to the original speed data (model aligned speed vs. raw speed: p = 1.17e-11, two-sided Wilcoxon signed-rank test, n = 61 sessions across 11 mice). Dots are sessions, colored by mouse; black edges, sessions with the reward zone at location “A” or “B”, included for the analysis shown in Fig. 6. In (**d**–**f**), gray dashed line marks the unity line. **e**) Reward vs. omission (RO) index calculated from the model-transformed neural data, shown for the same sessions in Fig. 6d, is highly correlated with the RO index calculated from the original neural data (two-sided Pearson correlations). The time warp model thus scales and aligns the firing rate curves between rewarded and omission trials but does not distort their relationship. **f**) MSE between trial-by-trial raw speed profiles before vs. after the reward switch. Dots are sessions, colored by animal as in (**d**); black edges, sessions included for analysis in Fig. 6. Speed MSE after the switch is significantly higher than before the switch (thus rewards and omissions are more difficult to compare on these trials): p = 1.07e-3, two-sided Wilcoxon signed-rank test, n = 61 sessions across 11 mice. **g**–**i**) Raw and model-aligned speed, neural activity, and RO indices from the same example cells shown in Fig. 6a–c but calculated from the trials after the reward switch. Data are shown as mean ± SEM across rewarded (black) and omission (magenta) “after” trials. Note preference of cells to fire more following rewards versus omissions, with the caveat that the speed profiles are more different than during the “before” trials. **j**) A linear mixed effects model shows no significant effect of switch day (β = 0.01, p = 0.46, two-sided Wald test), original MSE between mean speed on rewarded and omission trials (β = −0.001, p = 0.28), MSE of the time warp model fit (β = 0.001, p = 0.66), or the interactions of these terms (fixed effects) on the median RO index (random effects are mouse identity). Dots, median RO index per mouse (colored as in **d**) of the sessions included in Fig. 6e. Gray line and shading, model prediction ± 95% confidence interval. **k**) Reward vs. omission index across all switch days where the reward zone was at “A” or “B”, on the trials after the switch. In (**k**–**l**): **p < 0.0045 Bonferroni-corrected significance level, ***p < 0.001, ****p < 0.0001, from a two-sided, one-sample Wilcoxon signed-rank test against a 0 median. m3: median = 0.10, W = 5.01e3, p = 3.31e-9, n = 5 days, 197 cells; m4: median = 0.13, W = 2.10e3, p = 3.70e-6, n = 4 days, 126 cells; m7: median = 0.14, W = 4.21e3, p = 7.58e-8, n = 5 days, 177 cells; m11: median = 0.22, W = 106, p = 2.61e-5, n = 3 days, 41 cells; m12: median = 0.13, W = 2.57e4, p = 1.55e-13, n = 5 days, 419 cells; m13: median = 0.21, W = 377, p = 2.12e-4, n = 5 days, 58 cells; m14: median = 0.04, W = 9.84e3, p = 0.085, n = 3 days, 213 cells; m15: median = 0.08, W = 3.10e4, p = 1.98e-12, n = 5 days, 448 cells; m17: median = 0.05, W = 8.78e2, p = 0.049, n = 4 days, 69 cells; m18: median = 0.11, W = 2.21e4, p = 5.39e-15, n = 4 days, 401 cells; m19: median = 0.08, W = 3.03e3, p = 1.25e-5, n = 5 days, 144 cells. **l**) Reward vs. omission index across all switch days at any reward zone before the switch. m3: median = 0.13, W = 4.99e3, p = 4.86e-8, n = 5 days, 191 cells; m4: median = 0.15, W = 2.63e3, p = 7.11e-12, n = 5 days, 165 cells; m7: median = 0.09, W = 4.44e3, p = 2.08e-3, n = 4 days, 157 cells; m11: median = 0.20, W = 418, p = 3.96e-6, n = 6 days, 68 cells; m12: median = 0.13, W = 4.66e4, p = 1.96e-13, n = 7 days, 541 cells; m13: median = 0.10, W = 902, p = 0.021, n = 5 days, 72 cells; m14: median = 0.09, W = 3.35e4, p = 9.13e-9, n = 6 days, 442 cells; m15: median = 0.10, W = 7.77e4, p = 5.85e-10, n = 7 days, 656 cells; m17: median = 0.11, W = 3.73e2, p = 0.017, n = 3 days, 49 cells; m18: median = 0.14, W = 7.63e4, p = 1.63e-30, n = 7 days, 765 cells; median = 0.13, W = 3.24e3, p = 1.32e-7, n = 6 days, 158 cells. Source data

First, we tested whether RR firing following the reward was locked to the animal’s running speed rather than the expected reward location. We examined RR activity following the reward zone start on rewarded trials versus omission trials with comparable running speed. To align the running speed profiles across rewarded and omission trials, we fit a time warping model^36^ on the speed data and applied this time-warped transformation to the neural data (Methods and Extended Data Fig. 8a–f). We then computed a ‘reward versus omission’ index to quantify the difference in each cell’s activity following rewards versus omissions (Extended Data Fig. 8e).

**Fig. 6:**
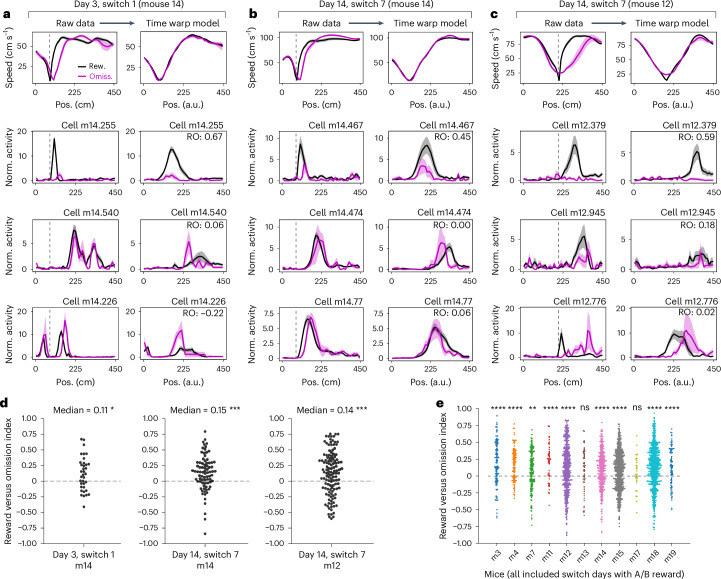
RR coding on rewarded versus omission trials, with a population preference for reward. **a**–**c**, Running speed (first row) and three RR example cells (second to fourth rows; activity normalized to each cell’s session mean) from trials before the reward switch. Vertical gray dashed line, reward zone start. Right columns: data transformed by the time warp model fit to the trial-by-trial speed. Data are shown as means across rewarded (rew.; black) and omission (omiss.; magenta) trials; shading, s.e.m. ‘RO’ (reward versus omission index) reflects the difference in model-transformed activity between rewarded and omission trials (positive, higher activity on rewarded trials; negative, higher activity on omission trials). Note that cell m14.474 (**b**) and cell m12.379 (**c**) are also in Fig. 2b. **d**, Reward versus omission index for the RR cell population with peak firing following the reward zone start, in the session shown in **a** (left), **b** (middle) and **c** (right). Each dot is a cell. *P < 0.05, ***P < 0.001, two-sided, one-sample Wilcoxon signed-rank test against a 0 median. Switch 1, m14: *W* = 183, *P* = 0.018, *n* = 36 cells; switch 7, m14: *W* = 721, *P* = 2.69 × 10^−5^, *n* = 79 cells; switch 7, m12: *W* = 2,358, *P* = 3.70 × 10^−4^, *n* = 122 cells. **e**, Reward versus omission index across all switch days where the reward zone was at ‘A’ or ‘B’ before the switch, for the RR cell population with peak firing following the reward zone start within each mouse (*n* = 11 mice). Each dot is a cell, colored by mouse. Significance is set at *P* < 0.0045 with Bonferroni correction for multiple comparisons (reported *P* values are uncorrected). **P < 0.0045, ****P < 0.0001, two-sided, one-sample Wilcoxon signed-rank test against a 0 median. m3, median = 0.14, *W* = 1.50 × 10^3^, *P* = 4.66 × 10^−7^, *n* = 3 days, 114 cells; m4, median = 0.20, *W* = 1.03 × 10^3^, *P* = 5.93 × 10^−10^, *n* = 3 days, 112 cells; m7, median = 0.09, *W* = 4.44 × 10^3^, *P* = 2.08 × 10^−3^, *n* = 4 days, 157 cells; m11, median = 0.23, *W* = 194, *P* = 5.32 × 10^−5^, *n* = 4 days, 48 cells; m12, median = 0.09, *W* = 3.41 × 10^4^, *P* = 4.40 × 10^−8^, *n* = 5 days, 441 cells; m13, median = 0.09, *W* = 523, *P* = 0.13, *n* = 3 days, 52 cells; m14, median = 0.11, *W* = 1.39 × 10^4^, *P* = 3.74 × 10^−8^, *n* = 4 days, 296 cells; m15, median = 0.09, *W* = 4.88 × 10^4^, *P* = 1.30 × 10^−7^, *n* = 5 days, 516 cells; m17, median = 0.03, *W* = 208, *P* = 0.20, *n* = 2 days, 33 cells; m18, median = 0.14, *W* = 4.86 × 10^4^, *P* = 1.18 × 10^−20^, *n* = 5 days, 590 cells; m19, median = 0.14, *W* = 1.02 × 10^3^, *P* = 9.67 × 10^−5^, *n* = 3 days, 88 cells. Source data

RR cells exhibited heterogeneity in their firing preference following rewards versus omissions (Fig. 6). Some fired at the same position relative to reward regardless of running speed (Fig. 6a,b, middle, and 6c, top and middle), while others fired relative to a particular phase of the speed profile (Fig. 6a,b, top and bottom). Notably, activity level often differed between rewarded and omission trials (Fig. 6a,b, top, and 6c, top and middle). At the population level, the RR cells fired significantly more following rewards compared to omissions, both before the reward switch (Fig. 6d,e) and after (Extended Data Fig. 8g–i,k). Thus, the RR population signaled information about the presence of past reward (Fig. 6e and Extended Data Fig. 8j–l).

**Fig. 7:**
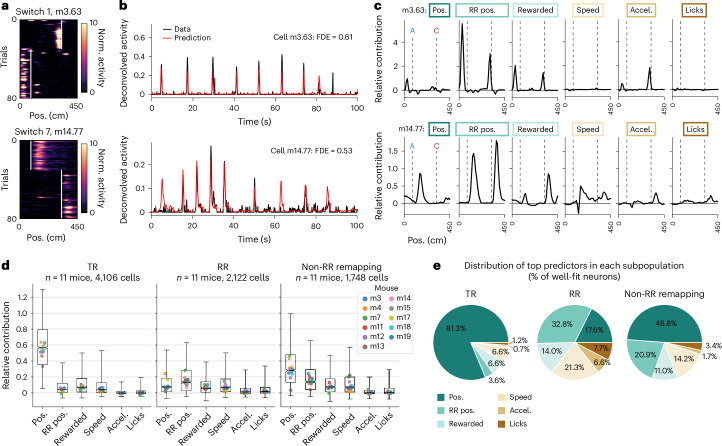
A GLM confirms encoding of RR position in the RR population. **a,** Example spatial firing maps of two RR cells that were well-fit by the GLM, on the first switch day (top) and last switch day (bottom). White lines, reward zone starts. Note that cell m14.77 (bottom) is also shown in Fig. 6b, bottom. **b**, Deconvolved calcium activity (black) and GLM prediction (red) of held-out test data for the two example cells shown in **a**. Fraction of Poisson deviance explained (FDE) of the test data is reported as a measure of model performance. **c**, Relative contribution (Methods), or encoding strength, for each variable included in the GLM, binned by track position for the two example cells shown in **a**. Task variables are ‘pos.’, linear track position; ‘RR pos.’ RR position; and ‘rewarded’, whether the animal was rewarded as a function of position (that is, a binary that stays high from the reward delivery time to the end of the trial). Movement variables are speed, acceleration (accel.) and licking (see Extended Data Fig. 9a for implementation). Relative contribution is calculated from an ablation procedure where each variable is individually removed from the full model (Methods). Gray dashed lines mark the start of the two reward zones ‘A’ and ‘C’ in each session, also indicated in **a**. Boxes correspond to color coding in **e**. Note that RR position provides the maximum relative contribution in the firing fields of both example cells, including m14.77, which fired equivalently on rewarded and omission trials before the switch (Fig. 6b). **d**, Distributions of relative contribution of each variable in the TR, RR and non-RR remapping subpopulations across animals. Boxes, interquartile range; whiskers, 2.5th to 97.5th percentile; horizontal line of each box, median; notches, confidence interval around the median computed from 10,000 bootstrap iterations. Colored dots, medians of each individual mouse. **e**, Distributions of top predictor variables (maximum relative contribution) for individual cells, shown as percentage of cells well-fit by the full GLM within each subpopulation; same *n* as in **d**. Source data

**Extended Data Fig. 9:**
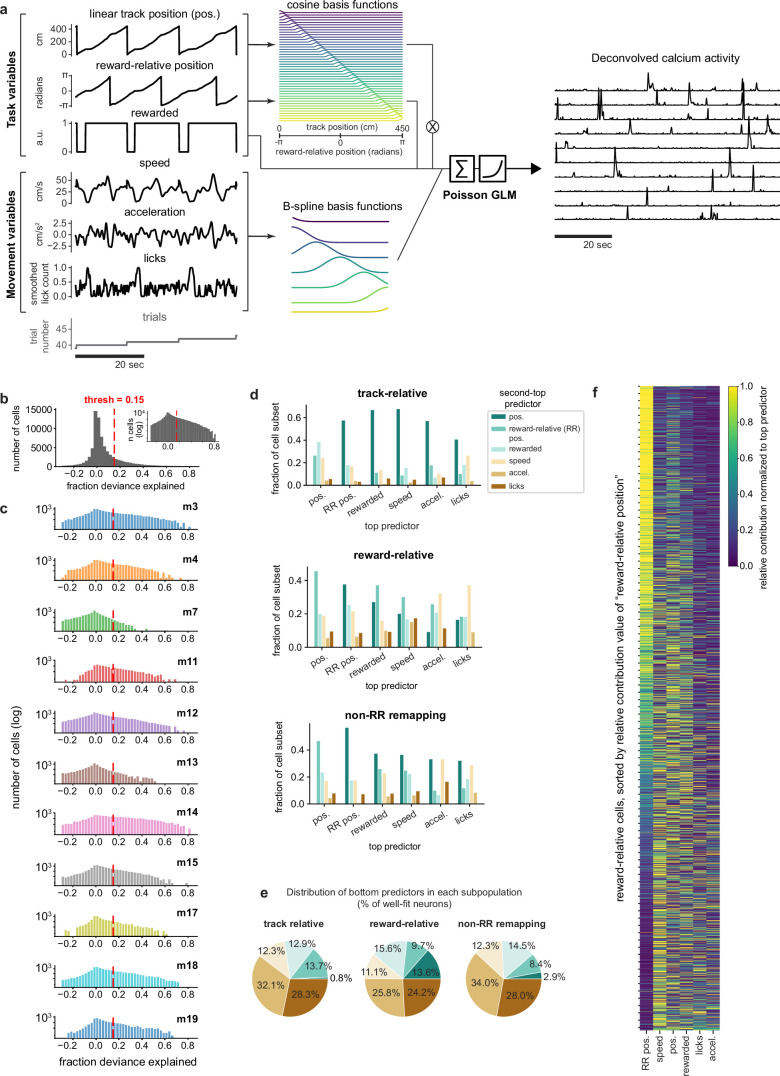
Generalized linear model (GLM) implementation and additional quantification. **a**) Schematic of the Poisson GLM to predict deconvolved calcium activity from task and movement variables. Linear track position and reward-relative position are both transformed into cosine basis functions tiling the space in each set of coordinates (45 cosine bumps for the 45 10-cm spatial bins used). A binary representing whether reward was received on each trial (“rewarded”) is multiplied with the linear track position basis functions to represent the interaction between reward received and position. Speed, acceleration, and smoothed lick count are quantile-transformed into B-splines which smoothly tile the ranges within each animal. Trial identities are used to group data for the training, cross-validation, and test sets (see Methods). **b**) Quantification of model performance (fraction deviance explained [FDE] on test data) across all switch animals and cells. Inset shows the histogram on a logarithmic scale. Red dashed line, fit threshold of 0.15 FDE used to select neurons for further analysis. Number and percentage of cells, pooled across all switch days, that exceeded the threshold: 11935/73512 (16%) of all cells, 11605/35386 (33%) of place cells from n = 11 mice. **c**) Quantification of model performance within each animal, shown on a logarithmic scale. Number and percentage of cells exceeding the FDE threshold: m3: 1549/6661 (23%) of all cells, 1425/2765 (52%) of place cells; m4: 1346/7600 (18%) of all cells, 1283/3583 (36%) of place cells; m7: 75/7367 (1%) of all cells, 72/2488 (3%) of place cells; m11: 337/1325 (25%) of all cells, 331/831 (40%) of place cells; m12: 1412/9286 (15%) of all cells, 1386/4998 (28%) of place cells; m13: 132/4808 (3%) of all cells, 124/1304 (10%) of place cells; m14: 2335/5487 (43%) of all cells, 2303/4086 (57%) of place cells; m15: 1392/9129 (15%) of all cells, 1386/5266 (26%) of place cells; m17: 506/4879 (10%) of all cells, 503/1889 (27%) of place cells; m18: 2215/13255 (17%) of all cells, 2177/6503 (33%) of place cells; m19: 636/3715 (17%) of all cells, 615/1723 (36%) of place cells. **d**) Distributions of second-top predictors for each subset of cells according to their top predictor, based on relative contribution of each variable computed by the model ablation method. In (**d**, **e**): n = 11 mice, 4106 track-relative cells, 2122 reward-relative cells, 1748 non-RR remapping cells. **e**) Distributions of bottom predictor variables (minimum relative contribution) for individual cells within each subpopulation, shown as % of well-fit cells in the subpopulation. **f**) All reward-relative cells well-fit by the GLM, sorted by their relative contribution of reward-relative position. Each row (each cell) is normalized by the contribution of its top predictor (n = 2122 reward-relative cells, 11 mice).

Second, we implemented a generalized linear model (GLM)^37^ to dissociate the contribution of task variables (linear track position, RR position and whether the animal was rewarded on each trial) versus movement variables (speed, acceleration and licking) to the deconvolved calcium activity of each cell (Extended Data Fig. 9a and Methods). We trained the GLM using fivefold cross-validation and tested its prediction on the activity of each neuron in held-out trials (Fig. 7a,b and Extended Data Fig. 9b,c). Only well-fit neurons (fraction deviance explained > 0.15)^37^ were included in further analysis (33% of place cells across 11 mice and seven switch days; TR cells, 4,106 out of 7,027; RR cells, 2,122 out of 5,979; non-RR remapping cells, 1,748 out of 4,314; Extended Data Fig. 9b–c).

We then performed a model ablation procedure to measure the relative contribution of each predictor variable to the activity of the TR, RR (Fig. 7c) and non-RR remapping subpopulations that were well-fit by the full model (Methods). We found that linear track position provided the highest relative contribution for the TR population (Fig. 7d). We next quantified the fraction of cells for which each variable was the top predictor, agnostic of its contribution score. Track position was the top predictor for 81.3% of TR cells (Fig. 7e), confirming their characterization as place cells locked to the spatial environment. The non-RR remapping cells were best predicted by linear track position followed by RR position (Fig. 7e and Extended Data Fig. 9d), consistent with their recruitment to the RR population over experience (Fig. 5). For RR cells, RR position provided the highest relative contribution at the population level (Fig. 7d) and the top predictor for 32.8% of RR cells, followed by speed (21.3%), linear track position (17.6%) and the receipt of reward (14.0%), with minimal contributions of acceleration and licking (Fig. 7e). RR position was also the second-top predictor among cells best predicted by speed, track position and reward (Extended Data Fig. 9d,e). Overall, ~56% of RR neurons well-fit by the GLM had RR position as a first or second predictor, yielding ~9% of place cells. Notably, RR position was coded in a gradient of strengths across the population compared to the other variables, consistent with mixed selectivity (Extended Data Fig. 9f). Although movement covariates are challenging to fully disentangle from progression through the task, both the GLM and analysis of rewarded versus omission trials provide evidence that reward and RR position are strong predictors in a population code for the animal’s experience around reward.

### RR cell activity often updates before behavior

**Fig. 8:**
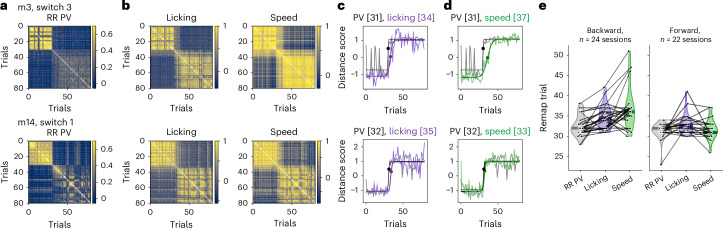
RR remapping often precedes changes in behavior. **a**, Trial-by-trial correlation matrix of the RR neural population vector (PV) (two example mice and sessions, top and bottom rows). Color scales show correlation in **a** and **b**. Reward zone switch occurs at trial 30. **b**, Trial-by-trial correlation matrix of the spatially binned licking behavior (left) and running speed (right) from the same two sessions as in **a**. **c**, Distance score indicating how close the trial-by-trial RR PV activity (gray) and licking (light purple) is to its *k*-means cluster centroid (*k* = 2) before versus after the reward switch (Methods). Black and dark purple lines, sigmoidal fits of PV and licking distance scores, respectively; dots, inflection point or ‘remap trial’, also listed in square brackets. **d**, Distance score for RR PV (gray; repeated from **c** for each mouse) and speed (light green) with sigmoidal fits (black and dark green). Remap trial for each is listed in square brackets. **e**, Remap trials of the RR PV precede the remap trials of licking and speed when the reward is moved backward on the track (left), and slightly precede licking but are synchronized with speed when the reward is moved forward on the track (right). Dots are sessions (*n* = 10 mice included); lines connect the same session across PV, licking and speed. Significant fixed effects from a linear mixed-effects model, with PV remap trial as the reference category and mice as random effects (*P* values from two-sided coefficient *t*-tests with Benjamini–Hochberg correction for multiple comparisons): licking – PV, *β* = 2.125, *P* = 0.042; speed – PV, *β* = 4.417, *P* = 9 × 10^−6^; interaction of forward switch direction and (speed – PV), *β* = −3.871, *P* = 0.011, indicating reduced lag between PV and speed in forward switch sessions versus backward. Non-significant fixed effects: switch direction (forward − backward) for PV, *β* = −1.436, *P* = 0.19; interaction of forward switch direction and (licking – PV), *β* = −0.398, *P* = 0.76; switch day, *β* = 0.157, *P* = 0.32. Independent linear mixed-effects models for backward and forward sessions with licking as the reference confirmed these results and that licking precedes speed only with backward switches (backward: PV – lick, *β* = −2.125, *P* = 0.018; speed – lick, *β* = 2.292, *P* = 0.018; forward: PV – lick, *β* = −1.727, *P* = 0.032; speed – lick, *β* = −1.182, *P* = 0.10). Source data

Given the RR coding we observed independent of movement covariates, we wondered whether this code could signal a change in the animal’s internal estimate of reward contingency, which should update before the behavior changes. To examine the timing of RR remapping compared to changes in licking and running speed following a reward switch, we computed a distance score^38^ reflecting the trial-by-trial proximity of population vector activity to the pre-switch or post-switch ‘maps’ (Methods). We also applied this method to licking and speed, and then fit a sigmoid to the distance score of each variable. The sigmoid inflection point was defined as the ‘remap trial’ at which the neural activity or behavior reliably deviated from its pre-switch map (Fig. 8a–d).

**Extended Data Fig. 10:**
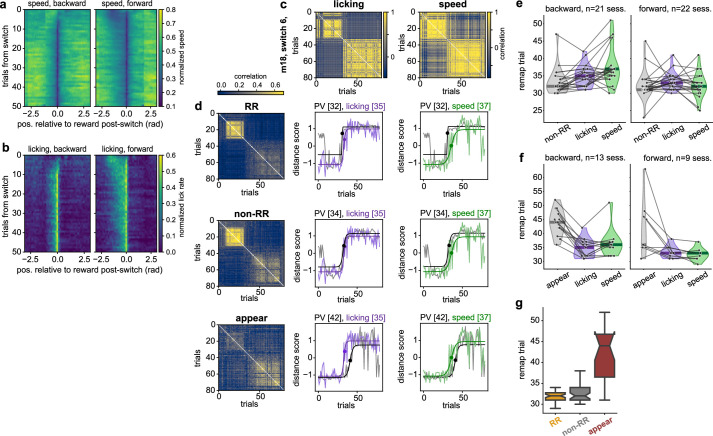
Coordination of remapping with behavior across hippocampal subpopulations. **a**) Mean speed post-reward-switch on each trial across backward (left; n = 24 sessions) and forward (right; n = 22 sessions) reward switches included for calculating reward-relative population remapping in Fig. 8. Note abrupt deceleration on the first post-switch trial for forward switches. Speed is normalized to the max of each session. **b**) Same as (**a**) but for mean licking on each trial. Lick rate is normalized to the max of each session. **c**) Trial-by-trial correlation matrix of the spatially binned licking behavior (left) and running speed (right) from example animal m18, day 12, switch 6. Reward zone switch occurs at trial 30. **d**) Left column: Trial-by-trial correlation matrix of the spatially binned population vector (PV) activity of each neuronal subpopulation from the same example mouse and day shown in (**c**). Color scale applies to all rows. Middle column: Distance score for PV activity (gray) and licking (light purple) with sigmoidal fit (PV: black, licking: dark purple). Dots indicate the inflection point or “remap trial”, also listed in square brackets. Right column: Same as middle but comparing the distance score for PV (gray, repeated from middle) to speed (green). **e**) PV remap trials for non-RR remapping cells compared to the remap trials of licking and speed. A linear mixed effects model (LMM) was fit to the remap trials as described in Fig. 8, with mice as random effects. In (**e**–**g**), p-values are from two-sided coefficient t-tests, with Benjamini-Hochberg correction for multiple comparisons. LMM fixed effects: lick – PV: β = 1.667, p = 0.30; speed – PV: β = 4.190, p = 0.0012; forward switch direction compared to backward, PV: β = −1.089, p = 0.52; forward switch direction x [lick – PV]: β = −0.621, p = 0.70; forward switch direction x [speed – PV]: β = −4.372, p = 0.020; switch day: β = 0.112, p = 0.63. **f**) Same as (**e**) but for appearing cells (limited to sessions in which 2 clusters could be identified in the PV activity). LMM fixed effects: lick – PV: β = −9.308, p = 6e-6; speed – PV: β = −7.462, p = 2.7e-4; forward switch direction compared to backward, PV: β = −3.222, p = 0.33; forward switch direction x [lick – PV]: β = 1.197, p = 0.69; forward switch direction x [speed – PV]: β = −1.538, p = 0.69; switch day: β = 0.218, p = 0.69. **g**) PV remap trial distributions for each subpopulation, pooled across backward and forward switches. Mean ± SD remap trial: RR = 31.91 ± 2.37; non-RR: 32.86 ± 3.91; appear: 43.0 ± 7.22. Boxes, interquartile range; whiskers, 2.5th to 97.5th percentile; horizontal lines, median; notches, median confidence interval from 10,000 bootstraps. Note smallest variance of RR cells. An LMM with fixed effects of subpopulation (compared to RR), switch direction, the interaction of switch direction and subpopulation, and switch day, with mice as random effects, shows significant effects only of subpopulation for appearing cells: non-RR – RR: β = 0.819, p = 0.57; appear – RR: β = 10.888, p = 2.2e-14; forward switch direction compared to backward: β = −1.40, p = 0.43; forward switch direction x [non-RR – RR]: β = 0.087, p = 0.96; forward switch direction x [appear – RR]: β = −2.752, p = 0.43; switch day: β = 0.174, p = 0.56. Source data

We found that remapping of the RR population was often synchronous with and frequently preceded changes in behavior (Fig. 8e). Neural remapping preceded changes in licking by approximately two trials regardless of whether the reward was moved backward or forward on the track (backward: 2.1 ± 0.6 trials (mean ± s.e.m.), neural before licking; forward: 1.7 ± 0.7 trials), but only preceded changes in speed when reward was moved backward (4.4 ± 1.1 trials, neural before speed) and was synchronous with speed when reward was moved forward (0.5 ± 0.5 trials before speed). This difference was caused by more abrupt reductions in running speed across the track when the reward was moved forward (Extended Data Fig. 10a,b), although the neural remap trial did not significantly change between switch directions (Fig. 8e). The remapping of the non-RR population also slightly preceded or was synchronous with behavior (Extended Data Fig. 10c–e), although timing was most consistent for the RR population (Extended Data Fig. 10g). By contrast, remapping of the appearing cells significantly followed changes in behavior (Extended Data Fig. 10c,d,f,g), consistent with the later formation laps we observed for appearing fields (Extended Data Fig. 3m). Altogether, these results raise the possibility that the RR population remaps sufficiently quickly at a trial-by-trial level to inform the animal’s behavior.

## Discussion

We reveal that the hippocampus simultaneously encodes an animal’s environmental location and its position relative to rewards through parallel, flexible population codes. RR coding spans the environment and is dissociable from spatial visual stimuli, consistent with the brain simultaneously monitoring experience relative to multiple reference frames^4,12,17,26,27^. Furthermore, we found that only the RR code, but not the TR spatial code, recruits more neurons with increasing task experience, suggesting that the hippocampus may selectively strengthen a representation of generalized events surrounding reward as animals learn the task requirement to estimate a hidden reward zone. These hippocampal ensembles are thus poised to support a stable spatial map while amplifying memory for behaviorally salient features such as reward.

We validated prior findings that a hippocampal subpopulation precisely encodes reward locations^22^. Here, we expanded upon this and other work^1,2,19–24^ in four key ways: first, reward-specific cells (±25 cm of reward^22^) comprise a central component of a more extensive RR sequence that extends hundreds of centimeters from one reward to the next, even across our teleportation period. This extended activity could support predictions about distant rewards in the future or past and generalize these computations to similar scenarios. Furthermore, we observed ordered remapping with respect to reward in contrast to the random reorganization observed with task disengagement^39^ or the absence of reward^40^. Second, the RR code is expressed dynamically at the population level rather than through a dedicated class of cells. We observed day-to-day turnover in membership of the RR population, consistent with representational drift, which is characterized by changing single-neuron spatial tuning^28,29^ despite preserved population-level coding^28^. Moreover, we did not observe any bias in the CA1 sublayer distribution of either subpopulation, in contrast to previous work^34,35^. In addition, RR and TR properties were expressed in individual place fields, raising the possibility that CA1 synapses driving distinct fields undergo independent plasticity. Together, these findings indicate a robust network code anchored to reward within a day, despite day-to-day flexibility. Third, more RR neurons are recruited as anticipatory behavior becomes more robust, increasing the field density around reward with the animal’s licking precision. Cross-day coding stability is also enhanced near reward, consistent with previous work^41^. However, by dissociating reward from the spatial environment, we revealed that this stability is specific to RR rather than spatial coding: TR fields initially close to reward were less stable across days and more likely to become RR. Interestingly, RR field density is highest following the reward zone start, where animals were most likely to get a reward, consistent with an increasingly robust hidden state estimation^6,42,43^ to accurately predict where reward is located. We further established that past reward influences this activity following the reward site, complementing recent findings of hippocampal action codes preceding reward^44^. Fourth, we found that RR population remapping tended to precede the animal’s update in behavioral strategy at the trial-to-trial level. Consistent with a role for hippocampal activity in eliciting reward-seeking actions^25^, our results suggest that the RR code updates sufficiently quickly to inform behavior, although future work will be required to test this possibility.

Linear VR paradigms constrain hippocampal firing to one dimension, potentially enhancing our detection of sequential fields relative to reward. In freely moving scenarios, the RR code may split to dissociate different goal locations over learning^21,23,45^ and probably integrates directional and route information in 2D^46–48^, similar to hippocampal encoding of goal vectors in freely flying bats^49^. Our results suggest that these individual tuning changes may reflect a coordinated network state representing the animal’s entire experience relative to reward. However, a limitation of linear VR paradigms is the inextricable link between anticipatory behaviors, such as slowing down, and RR position. Although we found that movement dynamics influence but do not dominate activity in the RR population via our GLM, further work will be required to fully disentangle which aspects of reward-seeking behavior are encoded in the hippocampus.

Prior work has inconsistently reported over-representation of reward^1,2^. Possible reasons for these discrepancies include: (1) the interaction between reward probability^50^ and whether reward is received at the goal location versus elsewhere^51^ (our ~85% reward probability resulting from the omission trials may have increased over-representation at the goal location, consistent with the effect of uncertain rewards^50^); (2) the ability to disentangle reward experience from other sensory cues (our VR dissociated the hidden reward from visual, olfactory and tactile stimuli); (3) the role of experience, evident in the gradual growth of over-representation over learning, although this density may dissipate in highly trained animals over longer time periods; and (4) the degree of movement stereotypy, as described above. These factors probably increased our ability to detect reward over-representation in the RR population.

Our work supports proposals that the hippocampus generates sequential activity^7,14,52^ anchored to salient reference points in experience^4^. At the sub-second timescale, theta sequences organized on the ~8 Hz theta rhythm^1^ are important for learning rewarded trajectories^53^ and representing possible current or future scenarios^54^. These sequences may alternate representations of reward zone configurations following a reward switch^55^. In addition, replay events during immobility may link the reward experience to updated locations in space^1,53^. Future studies using electrophysiology will be needed to explore these possibilities, given the slower temporal dynamics of calcium imaging.

A network of interacting brain regions is poised to inform RR coding. These include neuromodulatory inputs from the locus coeruleus^56^ and ventral tegmental area^40^, prefrontal cortical activity that encodes the animal’s progression towards goals^57,58^ and entorhinal cortical inputs that contribute to the hippocampal reward over-representation^19,59,60^. The inputs that drive RR coding in the hippocampus remain to be investigated.

## Methods

### Subjects

All procedures were approved by the Institutional Animal Care and Use Committee at Stanford University School of Medicine. C57BL/6J mice (seven males, seven females) were acquired from Jackson Laboratory. Mice were housed in a transparent cage (Innovive) in groups of five same-sex littermates before surgery, with access to an in-cage running wheel for at least 4 weeks. After surgery, mice were housed in groups of one to three same-sex littermates, with all mice per cage implanted. All mice were kept on a 12 h light–dark schedule, with experiments conducted during the light phase, and housed at ~22 °C and ~40–45% humidity. Mice were ~2.5–4.5 months of age at the time of surgery (weighing 18–31 g). Before surgery, animals had ad libitum access to food and water, and ad libitum access to food throughout the experiment. Mice were excluded from the study if they failed to perform the behavioral pre-training task described below.

### Surgery for calcium indicator expression and imaging window implants

Following previously established procedures for 2P imaging of CA1 pyramidal cells^6^, a 3 mm diameter, ~1.3 mm long stainless steel imaging cannula (McMaster) was affixed to a circular cover glass (Warner Instruments, number 0 thickness, 3 mm diameter; Norland Optics, number 81 adhesive). During the cannula implantation procedure, animals were anesthetized through intraperitoneal injection of ketamine (~85 mg kg^−1^) and xylazine (~8.5 mg kg^−1^), maintained with inhaled 0.5–1.5% isoflurane and oxygen at a flow rate of 0.8–1 l min^−1^ using a standard isoflurane vaporizer. Before surgery, animals received a subcutaneous administration of ~2 mg kg^−1^ dexamethasone and 5–10 mg kg^−1^ Rimadyl (to reduce inflammation and promote analgesia, respectively). An initial hole was drilled at the viral injection site targeting the left dorsal CA1 (antero-posterior (AP), −1.94 mm; medio-lateral (ML), −1.10 to −1.30 mm), and an automated Hamilton syringe microinjector (World Precisions Instruments) with a 35-gauge needle was used to inject 500 nl adenovirus (AAV) at 50 nl min^−1^ in the CA1 pyramidal layer (DV, −1.33 to −1.37 mm), to express the genetically encoded calcium indicator GCaMP under the pan-neuronal synapsin promoter (AAV1-Syn-jGCaMP7f-WPRE, AddGene, viral prep 104488-AAV1, titer 2 × 10^12^). The needle was left in place for 10 min to allow for virus diffusion.

A 3 mm diameter circular craniotomy (center: AP, −1.95 mm; ML, −1.8 to −2.1 mm; avoiding the midline suture) was then performed over the left hippocampus using a robotic surgery drill for precision (Neurostar). During drilling, the skull was kept moist with cold sterile artificial cerebrospinal fluid. The dura was then delicately removed using a bent 30-gauge needle. To access CA1, the cortex overlying the hippocampus was aspirated with continuous irrigation of ice-cold, sterile artificial cerebrospinal fluid until the fibers of the external capsule were clearly visible and left intact. Following hemostasis, the imaging cannula was lowered into the craniotomy until the cover glass lightly contacted the external capsule. To minimize structural distortion and image tangential to the CA1 pyramidal layer, the cannula was positioned at a ~10° roll angle relative to horizontal. Cyanoacrylate adhesive was used to affix the cannula in place and cover the exposed skull surface, which was pre-scored with a number 11 scalpel before the craniotomy to provide increased surface area for adhesive binding. A headplate featuring a left-offset 7 mm diameter beveled window and lateral screw holes for attachment to the imaging rig was positioned over the imaging cannula at a matching 10° angle and cemented to the skull using Metabond dental acrylic dyed black with India ink or black acrylic powder. Following the procedure, animals were administered 1 ml of saline and 10 mg kg^−1^ of Baytril antibiotic, then placed on a warming blanket for recovery. A minimum 10-day recovery period was required before initiation of head-fixation and VR training.

### Histology

Mice were deeply anesthetized and administered an overdose of Euthasol, then perfused transcardially with PBS followed by 4% paraformaldehyde in PBS. Brains were removed and post-fixed in paraformaldehyde for 24 h, followed by incubation in 30% sucrose in PBS for >4 days. Next, 50 µm coronal sections were cut on a cryostat, mounted on gelatin-coated slides and coverslipped with DAPI mounting medium (Vectashield). Histological images were taken on a Zeiss widefield fluorescence microscope.

### VR design

All VR tasks were designed and operated using custom code written for the Unity game engine (https://unity.com, v.2020.3.19), on a separate computer from the calcium imaging acquisition computer. Virtual environments were displayed on three 24-inch LCD monitors surrounding the mouse at 90° angles relative to each other. The VR behavior system included a rotating fixed-axis cylinder to serve as the animal’s treadmill and a rotary encoder (Yumo) to read axis rotations and record the animal’s running. A capacitive lick port, consisting of a feeding tube (Kent Scientific) wired to a capacitive sensor, detected licks and delivered sucrose water reward (5% sucrose w/v) through a gravity solenoid valve (Cole Palmer). Two separate Arduino Uno microcontrollers operated the rotary encoder and lick detection system. Behavioral data were sampled at approximately 50–75 Hz, matching the VR frame rate. Both the start of the VR task as well as each VR frame were synchronized with the ~15.5 Hz sampled imaging data by Unity-generated TTL pulses from an Arduino to the imaging computer.

### Behavioral training and VR tasks

#### Handling and pre-training

After recovery from surgery, the mice were handled for 2–3 days for 10 min each day, then acclimated to head-fixation on the cylindrical treadmill for approximately 15, 30 and 60 min over each of 3 days. To motivate behavior, mice were water-restricted to 85% or higher of their baseline body weight. Mice were weighed daily to monitor health and underwent hydration assessments (by skin tenting). Mice were acclimated to licking operantly for sucrose water rewards delivered at a minimum of 2 s intervals for 1–2 days. The water delivery system was calibrated to release ~4 μl of liquid per drop. The volume of water consumed during the experiment was measured, and supplemental water was supplied if needed up to a total of ~0.045 ml g^−1^ day^−1^ (typically 0.8–1 ml day^−1^, adjusted to maintain body weight), before animals were returned to their home cage each day.

Once acclimated to the lick port, mice were pre-trained on a ‘running’ task on a 350 cm virtual linear track with random black and white checkerboard walls and a white floor to collect a cued reward. The reward location was marked by two large gray towers, initially positioned 50 cm down the track. The mouse was rewarded for licking within 25 cm of the towers, otherwise, an automatic reward was given at the towers. The towers then disappeared and quickly reappeared further down the track. Covering the distance to the current reward in under 20 s increased the inter-reward distance by 10 cm, but if the mouse took longer than 30 s, the distance decreased by 10 cm, with a maximum reward location of 300 cm. Upon consistent completion of 300 cm distance in under 20 s, the automatic reward was turned off, requiring the mice to lick to receive reward. At the end of the track, the mice passed through a black curtain and into a gray ‘teleport zone’ (50 cm long) that was equiluminant with the VR environment before re-entering the track from the beginning. Once mice were reliably licking and completing 200 laps of the pre-training track within a 30–40 min period, they were advanced to the main task below and imaging (mean ± s.d., 5.8 ± 1.9 days of running pre-training across 14 mice).

#### Hidden reward zone task

The main ‘switch’ task involved two virtual environments similar to those previously used to study hippocampal remapping^6^, each with visually distinct features from the pre-training environment. Both environments consisted of a 450 cm linear track, with two colored towers (green in ENV 1, blue in ENV 2) and two patterned towers along the walls. Environments were further distinguished by the frequency of diagonal wall gratings (low, ENV 1; high, ENV 2) and color of the sky (dark gray, ENV 1; light gray, ENV 2). The reward zone was a ‘hidden’, unmarked 50 cm span at one of three possible locations along the track, each equidistantly spaced between the towers to control for proximity of each reward to visual landmarks^20,32,33^: zone A, 80–130 cm; zone B, 200–250 cm; zone C, 320–370 cm. Only one reward zone was ever active at a time. Reward was randomly omitted on approximately 15% of trials, determined by a random number generator for each trial. Each lap terminated in a black curtain following by a ‘teleport period’ that began with an unchanging gray display for a randomly jittered amount of time (‘jitter period’) between 1 and 5 s (5–10 s on trials following reward omissions or trials in which the mouse did not lick in the reward zone), after which the gray display began to move again for 50 cm giving the appearance of a gray ‘tunnel,’ before the mouse re-entered the virtual environment at 0 cm.

Each mouse encountered a different starting reward zone and sequence of reward zone switches, counterbalanced across mice (*n* = 11 mice). Most mice began the task in ENV 1 (*n* = 9 mice; two mice (m17 and m18) began in ENV 2). An initial reward zone (for example, A as in Fig. 1e) was acquired for days 1–2 of the task. On day 3 (switch one), the zone was moved after 30 trials to one of the two other possible locations on the track (for example, B). On the first ten trials of any new condition, including the first day and each subsequent switch, the reward was automatically delivered at the end of the zone if the mouse had not yet licked within the zone, to signal reward availability. Otherwise, the reward was delivered at the first location within the zone where the mouse licked. After these ten trials, a reward was operantly delivered for licking within the zone, and we observed that the mice often started licking before ten trials elapsed (Extended Data Fig. 1a–c). The new reward zone was maintained on day 4. On day 5 (switch two), the zone was moved to the third possible reward zone (for example, C), maintained on day 6 and moved back to the original location on day 7 (for example, A). On day 8, the reward zone switch coincided with a switch into the novel environment, where the sequence of zone switches was then reversed on the same day-to-day schedule for a total of 14 days (Fig. 1 and Extended Data Fig. 1). Each switch occurred after 30 trials.

An additional ‘fixed-condition’ cohort (*n* = 3 mice) experienced only ENV 1 and one fixed reward location throughout the 14 days (Extended Data Fig. 1d,f).

We targeted 80–100 trials per session with simultaneous 2P imaging (described below), with one imaging session per day (mean ± s.d., 80.5 ± 7.4 trials across 14 mice, all imaging days). The session was terminated early if the mouse ceased licking or running consistently and/or if the imaging time exceeded 50 min. Before the imaging session, mice were provided 30 ‘warm-up’ trials using the task and reward zone from the previous day to re-acclimate them to the VR setup (using the pre-training environment on day 1 of imaging). Following each daily imaging session, mice were given another ~100 training trials without imaging on the last reward zone seen in the imaging session until they acquired their daily water allotment.

### 2P imaging

We used a resonant-galvo scanning 2P microscope from Neurolabware with Scanbox software (https://scanbox.org, v.4.7) operated through MATLAB (Mathworks) to image the calcium activity of CA1 neurons. Excitation was achieved using 920 nm light from a tunable output femtosecond pulsed laser (Coherent Chameleon Discovery). Laser power modulation was accomplished with a Pockels cell (Conoptics). The typical excitation power, measured at the front face of the objective (Leica ×25, 1.0 NA, 2.6 mm working distance), ranged from 15–68 mW for mice m2–m7, 50–100 mW for m10 and m11, and 13–40 mW for m12–19. The task was imaged starting from day 1 for all mice except m11, for whom imaging started on day 3 owing to lower viral expression. For most animals and sessions, to minimize photodamage and photobleaching, the Pockels cell was used to reduce laser power to minimal levels between trials (during the teleport period), except for mice m11–m14 on task days 1, 7, 8 and 14, and mice m15–m19 on day 1 and all switch days, for which laser power was maintained and imaging continued throughout the teleport period. Photons were collected using Hamamatsu gated GAsP photomultiplier tubes (part H11706-401 for Neurolabware Microscopes). The imaging field of view (FOV) was collected at 1.0 magnification with unidirectional scanning at ~15.5 Hz, resulting in an approximately 0.7 × 0.7 mm FOV (512 × 796 pixels). An electrically tunable lens was used to simultaneously image deep and superficial CA1 in two mice (m17 and m18), in two planes separated by ~27 µm, with frames bidirectionally imaged at ~31 Hz interleaved in the scan for a sampling rate of ~15.5 Hz per plane. In all mice, the same FOV was acquired each session by aligning to a reference image from previous days before the start of data acquisition, with the aid of the ‘searchref’ plugin in Scanbox. This allowed us to track single cells across days.

### Calcium data processing

The Suite2P software package (v.0.10.3)^61^ was used to perform *xy* motion correction (rigid and non-rigid) and identify putative cell regions of interest (ROIs). Manual curation eliminated ROIs containing multiple somata or dendrites, lacking visually obvious transients, suspected of overexpressing the calcium indicator or exhibiting high and continuous fluorescence fluctuation typical of putative interneurons. This approach yielded 155–2172 putative pyramidal neurons per session, owing to variation in imaging window implant quality and viral expression. In multi-plane imaged animals (see ‘2P imaging’), ROIs were identified separately per plane, but planes were pooled for all analyses except those in Extended Data Fig. 7. Custom code was used to follow individual ROIs across imaging sessions using the intersection over union of their pixels. The threshold for ROI matching was chosen algorithmically for each dataset such that the intersection over union for the best match for an ROI pair was always greater than the intersection over union for any second-best match.

To compute the Δ*F*/*F* (d*F*/*F*) for each ROI, baseline fluorescence was calculated within each trial independently using a maximin procedure with a 20 s sliding window. Limitation to individual trials both accounts for potential photobleaching over the session and avoids the teleport periods for sessions during which the laser power was reduced (see ‘2P imaging’). The value of d*F*/*F* was then calculated for each cell as the fluorescence minus the baseline, divided by the absolute value of the baseline, then smoothed with a two-sample (~0.129 s) s.d. Gaussian kernel. The activity rate was extracted by deconvolving d*F*/*F* with a canonical calcium kernel using the OASIS algorithm^62^ as used in Suite2p. We note that this deconvolution is not interpreted as a spike rate but rather as a method to eliminate the asymmetric smoothing of the calcium signal induced by indicator kinetics. Additional putative interneurons were detected for exclusion from further analysis by a Pearson correlation of >0.5 between their d*F*/*F* timeseries and the animal’s running speed, excluding 0.42 ± 0.85% of cells (mean ± s.d. across mice and days).

### Statistics and reproducibility

To avoid assumptions about data distributions, we used nonparametric tests or permutation tests in most cases, except when a Shapiro–Wilk test for normality confirmed that parametric tests were reasonable. In the main text, percentages of cells classified by remapping type are reported as mean ± s.d. out of all place cells identified on the specified days. Cells were excluded from certain analyses only if they did not meet pre-established criteria as ‘place cells’ (defined in ‘Place cell identification’), to ensure that included cells provided reliable information about position in the task. Averaged data in figures are shown as mean ± s.e.m. unless otherwise indicated. For the distributions of sequence positions over days in Fig. 4 and Extended Data Fig. 4, s.e.m. shading is omitted for clarity, but the variance for these distributions is accounted for in linear mixed-effects models to quantify these plots (see ‘Linear mixed-effects models’). For data displayed as violin plots, the shape is computed as a kernel density estimate to the bounds of the data, the solid center line marks the median and additional horizontal dashed lines mark the inner quartile.

All analyses were performed in Python (v.3.8.5). Linear mixed-effects models were performed using the mixedlm method of the statsmodels package (https://www.statsmodels.org/stable/mixed_linear.html). Pairwise repeated measures *t*-tests were run using the pingouin package (https://pingouin-stats.org) with a Holm step-down correction for multiple comparisons. Linear regressions and Wilcoxon signed-rank and rank-sum tests were performed using the SciPy (https://scipy.org) statistics module, with linear regression confidence intervals computed with the uncertainties package (https://pypi.org/project/uncertainties). Circular statistics were performed using Astropy (https://docs.astropy.org/en/stable/stats/circ.html), pycircstat^63^ (https://github.com/circstat/pycircstat) and circular–circular correlation code originally written to analyze hippocampal theta phase precession^64^ (https://github.com/CINPLA/phase-precession). The time warp models, GLM and factorized *k*-means were implemented from publicly available repositories (time warp, https://github.com/ahwillia/affinewarp; GLM, https://github.com/HarveyLab/GLM_Tensorflow_2; factorized *k*-means, https://github.com/ahwillia/lvl).

The number of mice to include was determined by having coverage of every possible reward sequence permutation (six sequences) with at least one mouse in the switch task. In addition, our mouse sample sizes were similar to those reported in previous publications^6,35^. Mice were randomly selected to experience the switch task (*n* = 11 mice) versus the ‘fixed-condition’ task in which the reward zone was not moved (*n* = 3).

### Estimation of anatomical location per ROI

AP and ML ROI coordinates were computed as the 2D center of mass of ROI pixels, relative to each FOV. In multi-plane imaged animals, ROIs were separated according to their deep or superficial imaging plane. To estimate deep-superficial (dorso-ventral (DV)) coordinates in single-plane animals, we acquired a *z*-stack scan after the day 14 task session that extended −100 µm below (−60 µm below in one mouse) to +100 µm above the FOV in the DV axis, in 2 µm steps with ~50 frames per step. Each step was independently motion-corrected as described above, and the mean of each step was used to compile the 3D *z*-stack array. We then took the AP projection of each ML slice of the *z*-stack (that is, looking at the side view of the *z*-stack). We minimum-filtered (4 × 0 pixel window, ML × DV) and smoothed (2 × 2 pixel s.d. Gaussian) each slice, then detected the most prominent dip in brightness in the DV axis to approximate the location of cell bodies, under the assumption that the GCaMP signal is typically absent in the nuclei and brighter in the surrounding neuropil. This yielded a per-pixel estimate of the depth of the pyramidal layer center from the dorsal surface of the *z*-stack, which we then smoothed (40 × 40 pixel s.d. Gaussian) to approximate the cell layer ‘curvature’ across the FOV. The curvature estimate was registered to the mean FOV of each imaging session. Given that the imaging plane transects this curvature, calculating the mean DV distance of each ROI (across pixels) from the curvature provides an estimate of where each ROI resides in the deep-to-superficial axis.

### Quantification of licking behavior

The capacitive lick sensor allowed us to detect single licks. A very small number of trials with erroneous lick detection from damage to the circuit were removed from subsequent licking analysis by setting these values to NaN (~0.65% of all imaged trials, *n* = 81 out of 12,376 trials removed across 11 switch mice). These trials were detected by >30% of the 0.0645 s imaging frame samples in the trial containing a cumulative lick count >2, as this would have produced a sustained lick rate ≥20 Hz. Remaining lick counts were converted to a binary vector and spatially binned at the same resolution as neural activity (10 cm bins), then divided by the time occupancy in each bin to yield a lick rate. We quantified licking precision over blocks of ten trials using an anticipatory lick ratio, computed as:

$$\rm{Lick}\; \rm{ratio}=\frac{\rm{Lic}\rm{k}_{\rm{in}}-\rm{Lic}\rm{k}_{\rm{out}}}{\rm{Lic}\rm{k}_{\rm{in}}+\rm{Lic}\rm{k}_{\rm{out}}}$$

where Lick_in_ is the mean lick rate in a 50 cm ‘anticipatory’ zone before the start of the reward zone and Lick_out_ is the mean rate outside of this zone and the reward zone. The reward zone itself is thus excluded to exclude consummatory licks (Extended Data Fig. 1e). A ratio of 1 indicates perfect licking only in the anticipatory zone, a ratio of −1 indicates licking only outside of this zone and a ratio of 0 indicates chance licking everywhere, excluding the reward zone.

### Place cell identification

For all neural spatial activity analyses, we excluded activity when the animal was moving at <2 cm s^−1^. To obtain an activity matrix of trials × position bins for each cell, we binned the 450 cm linear track into 45 bins of 10 cm each. We took the mean d*F*/*F* or deconvolved calcium activity on each trial within each position bin (note that taking the mean activity over time samples is equivalent to normalizing by the occupancy within a trial).

We defined place cells as cells with significant SI^65^ in either trial set 1 (pre-reward-switch) or trial set 2 (post-reward-switch) in a session. On days without a switch, we used the first and second halves of trials. For the SI, we used the deconvolved calcium activity as this reduces the asymmetry of the calcium signal. SI was calculated for calcium imaging^66^ as:

$${{\rm{SI}}}=\mathop{\sum }\limits_{i=1}^{N}{p}_{i}\frac{{f}_{i}}{f}{\rm{log}}_{2}\frac{{f}_{i}}{f},$$

where *p*_*i*_ is the occupancy probability in position bin *i* for the whole session, *f*_*i*_ is the trial-averaged activity per position bin *i* and *f* is the mean activity over the whole session, computed as the sum of *f*_*i*_ *×* *p*_*i*_ over all *N* bins. To determine *p*_*i*_, we calculated the per-trial occupancy (number of imaging samples) in each bin divided by the total number of samples per trial, then summed the occupancy probabilities across trials and divided by the total per session. To determine the significance of the SI scores, we created a null distribution by circularly permuting the position data relative to the timeseries of each cell, by a random amount between ~1 s and the length of the trial, independently on each trial. SI was calculated from the trial-averaged activity of each shuffle, and this shuffle procedure was repeated 100 times per cell. A cell’s true SI was considered significant if it exceeded 95% of the SI scores from all shuffles within an animal, pooled across cells (more stringent than comparing to the shuffle of each individual cell^67^). For plotting place cell firing over trials (for example, Fig. 2), deconvolved calcium activity was normalized to the mean of each cell within a session and smoothed with a 10 cm s.d. Gaussian.

### Spatial peak firing identification

To identify spatial peak firing of place cells, we used the position bin of the maximum unsmoothed, spatially binned d*F*/*F*, as this signal is the closest to the raw data, averaged across trials within a set (pre-switch or post-switch). There was no restriction to field boundaries, thus allowing cells to have multiple fields with a single identified peak.

### Trial-by-trial similarity matrices

Correlation matrices were computed using the spatially binned deconvolved activity on each trial, smoothed with a 20 cm s.d. Gaussian for single cells and a 10 cm s.d. Gaussian for population vectors. This resulted in a matrix, *A*, of *j* trials × *m* position bins per cell. For population vectors, single-cell matrices were horizontally concatenated such that *A* was *j* trials × *m* position bins × *n* cells. Each trial was *z*-scored across the position axis. For single cells, the trial-by-trial correlation matrix *C* was computed as: $C=\frac{1}{m-1}A{A}^{T}$; for population vectors: $C=\frac{1}{{mn}-1}A{A}^{T}$.

### Remapping category definitions

We defined the cell remapping types shown in Fig. 2 and Extended Data Fig. 2 as follows: TR, significant SI before and after the switch, with spatial peaks (see ‘Spatial peak firing identification’) ≤50 cm from each other before versus after; disappearing, significant SI before but not after the switch, with a mean spatially binned d*F*/*F* after that is less than the 50^th^ percentile of the per-trial mean d*F*/*F* before; appearing*,* significant SI after but not before, with a mean spatially binned d*F*/*F* after that is greater than 1 s.d. above the mean in trials before (appearing and disappearing cells that had sufficiently reliable activity despite their rate-remapping to be captured by the RR criteria below were removed from the appearing and disappearing groups); remap near reward, significant SI before and after, with spatial peaks ≤50 cm from the starts of both reward zones; and remap far from reward, significant SI before and after, not TR, with peaks >50 cm from the start of at least one reward zone.

### RR remapping

#### Quantification of RR remapping compared to chance

For stringent statistics on putative RR remapping (Fig. 2c–i and Extended Data Fig. 2g–n), we restricted the set of included place cells to require significant SI in the pre-switch and post-switch trial sets in each session. We converted the position timeseries of the animal to periodic coordinates, setting 0 cm on the track to −π and 450 cm to π. Spatial peaks for each cell were re-computed using binned d*F*/*F* at a periodic bin size of 2π / 45 (corresponding to ~10 cm). Putative TR cells were removed by excluding cells with spatial peaks within ~0.698 radians (50 cm) of each other before versus after the switch. The periodic coordinates were then circularly rotated to align the start of each reward zone at 0, to measure the signed circular distance of spatial peaks relative to the start of each reward zone. For scatter plots of spatial peaks in Fig. 2 and Extended Data Fig. 2, points are jittered by a random amount between −π / 100 and +π / 100 for visualization only. We measured the circularly wrapped difference between relative peaks after minus before the switch, creating a distribution that can be thought of as orthogonal to the unity line in Fig. 2d,f. We compared this distribution to a ‘random-remapping’ shuffle, generated by maintaining the pre-switch peak of each cell and circularly permuting its post-switch firing 1,000 times by a random offset of 0–44 bins. To define a candidate range of RR remapping variability, we computed the circular difference between the maximum and minimum bin of the distribution of true differences between RR peaks that exceeded the upper 95% of the shuffle, divided by two and averaged across switches. In an initial cohort of *n* = 7 mice, this produced a mean range of ±0.656 radians (~46.9 cm) around 0, which is captured by a 50 cm threshold given our 10 cm bin size. This threshold was subsequently used to identify candidate RR remapping cells (see below).

#### Criteria to define RR cells

As we observed variable remapping dynamics over trials after a reward switch (Fig. 2a,b and Extended Data Fig. 2a), which could affect SI scores, we relaxed the SI criterion for RR cells such that they were required to have significant SI either before or after the switch but not necessarily both. We next implemented two criteria to identify a robust subpopulation of RR cells. First, we identified place cells with peak spatial firing relative to the reward zone start within ~0.698 radians (50 cm) of each other before versus after a reward switch (within the orange candidate zone highlighted in Fig. 2d,f), allowing for some variability in RR precision. These cells could thus include ‘remap near reward’ and ‘remap far from reward’ cells (see ‘Remapping category definitions’). Second, to reduce the influence of noise in spatial peak detection and account for the shape of the firing fields, we cross-correlated the cells’ trial-averaged, spatially binned d*F*/*F* aligned to the reward zone pre-switch versus post-switch. We predicted that RR cells should have a peak cross-correlation close to zero on this reward-centered axis. We calculated a shuffle for the cross-correlation by circularly permuting the activity in each post-switch trial by a random offset between 1 and 45 bins 500 times, requiring that the real cross-correlogram had a peak exceeding the upper 97.5% confidence interval of the shuffle and that the offset of this peak was within five bins (~50 cm) of zero. This approach provides a metric for how stable each cell’s firing is relative to reward, identifying a subpopulation of cells whose fields were maximally Pearson-correlated in periodic coordinates relative to reward, in contrast to the TR cells, which were maximally correlated relative to the original linear track coordinates (Extended Data Fig. 2o–q). Place cells that passed both criteria were defined as RR.

### Relationship between distance run in the teleport zone and trial-to-trial variability

We measured the distance run in the teleport zone at the end of each trial by integrating the animal’s speed from the first frame in the teleport period to the last frame before re-entry to the start of the track. We then computed the trial-wise spatial peak error for each RR cell as the difference between the cell’s spatial peak location on each trial and its trial-averaged spatial peak location within either the trials before or after the reward switch (circular error converted back to cm). We next performed a Spearman correlation between the distance run during the teleport and the error on the following trial (Extended Data Fig. 2e,f).

### Individual place field analysis

#### Place field definition and inclusion criteria

Using the spatially binned, deconvolved activity smoothed with a 10 cm s.d. Gaussian, excluding activity from movement speeds <2 cm s^−1^, candidate place fields were identified as regions of ≥20 cm that exceeded 20% of the maximum of the trial-averaged activity^19^ (over the 30 pre-switch trials or the final 30 post-switch trials). To include a given field, the cell was required to be significantly active within the field boundaries in at least eight (>25%) of the 30 trials, where ‘significantly active’ was defined as the raw, un-binned deconvolved activity at those positions exceeding its mean +1 s.d. across the 30 trials. For analyses of place field properties before versus after a reward switch, cells were excluded that did not have significant SI or a significant field both before and after the switch. The fraction of cell remapping types meeting these criteria on specific days is reported in Extended Data Fig. 3c. For analyses of place field width and in-field activity rate, cells were further excluded if one of their fields overlapped the first or last spatial bin of the track, as this prevented accurate detection of field boundaries that extended into the teleport zone (which was not imaged for some animals; see ‘2P imaging’). Place field width was defined as the last minus the first position bin that crossed the initial 20% activity threshold.

#### Coordination of remapping between fields

We assessed the alignment of the center of mass of each field to RR or TR coordinates. We first confirmed that for cells with one field before and after the switch, the field center of mass replicated the robust RR and TR remapping patterns (Extended Data Fig. 3e) observed for spatial firing peaks (Fig. 2c,d,f). Next, for the subset of cells that had exactly two fields both before and after the switch, we computed the circular–circular correlation of the offsets between fields before versus after the switch (Extended Data Fig. 3f,g).

#### Formation lap identification and backwards shifting

To quantify the degree of backward and forward shifting of place fields following a reward zone switch, we first identified the formation lap (trial) of each field post-switch by adapting a previously established procedure^31^. Within the bounds of each trial-averaged field ±10 cm after the switch, we identified trials in which the post-switch spatially binned activity exceeded its mean +1 s.d. We then found the first five-trial window with at least three trials above this threshold and took the first active trial in that window as the formation lap^31^. To compute the field’s center of mass on each lap with maximum spatial resolution, we used the raw deconvolved activity and position within the bounds of the trial-averaged field. We then subtracted the center of mass on the formation lap from the center of mass of the field’s mean activity in the last 30 trials of the session to quantify the degree of shift (negative values indicate a backwards shift).

### Decoding of RR position

We implemented a previously developed decoder that uses circular–linear regression^38^ to predict the animal’s position in circular, RR coordinates from the deconvolved calcium event timeseries (at speeds of >2 cm s^−1^) of RR, TR or non-RR remapping neurons. The model ‘decode score’ at each timepoint *t* is given by $\cos ({{y}_{t}}-\hat{{y}}_{t})$, where ${y}_{t}$ is the animal’s true RR position and $\hat{{y}}_{t}$ is the predicted RR position. A score of one indicates perfect prediction and zero indicates random prediction. We performed tenfold cross-validation in which we trained the decoder on 90% of the data and tested it on a held-out 10%, either training and testing within the trials before the switch or training on one set (before) and testing on the other set (after). Figure 3a shows decoder performance from a single cross-validation fold, considering all data from an example session. To quantify decoding accuracy compared to chance, we next downsampled the data to match occupancy at each RR position bin (bin size 2π / 45, ~10 cm), either within trials before the switch or in the last 30 trials after the switch (thereby matching trial numbers before and after). We then repeated model training and testing with 100 shuffled datasets for each session and cell subpopulation. In each shuffle, the timeseries of each cell was independently circularly shifted by a uniform random amount up to the number of samples in the session minus one. In Fig. 3b, model performance is reported as a mean decode score over time samples and cross-validation folds compared to the mean of the shuffles for each session. To evaluate decoder performance across sessions, we *z*-scored the decoder score of each session to its shuffle and binned the *z*-score as a function of RR position, then averaged across animals (Fig. 3c).

### Sequence detection and quantification

For analysis of behavioral timescale sequential firing of neural subpopulations, we used the unsmoothed, spatially binned d*F*/*F* in circular track coordinates (not aligned to reward, although aligning to reward makes no difference for the circular sequence order). We sorted neurons by their peak firing positions from activity averaged over the odd trials before the switch. This sort was cross-validated^6^ by plotting the trial-averaged activity of the even trials before the switch (for example, left-hand columns of Fig. 4a, normalized to the mean activity of each cell within a session), then applying this sort to the trials after the switch. The sequence positions before versus after were then taken as the peak firing positions of the trial-averaged ‘even-before’ trials and the trial-averaged ‘after’ trials. Preservation of sequence order was quantified as the circular–circular correlation coefficient^64^ between the sequence positions before versus after. Although a *P* value can be directly computed for this correlation coefficient, its significance was further validated by randomly permuting the cell identities of the neurons after the switch and re-computing the correlation coefficient 1,000 times to obtain a null distribution. The observed correlation coefficient, rho, was considered significant if it was outside 95% of the null distribution using a two-tailed test. The *P* value was calculated as:

$$P=\frac{{n}_{\left|{{\rm{coef}}_{\rm{shuf}}}\right|\ge \left|{{\rm{coef}}_{\rm{obs}}}\right|}+1}{{N}_{\rm{perm}}+1},$$

where ${n}_{\left|{{\rm{coef}}_{\rm{shuf}}}\right|\ge \left|{{\rm{coef}}_{\rm{obs}}}\right|}$ is the number of shuffled coefficients with absolute values greater than or equal to the absolute value of the observed coefficient, and ${N}_{\rm{perm}}$ is the total number of shuffles. *P* < 0.001 indicates that the observed rho exceeded all 1,000 shuffles.

For sequences followed across days, we again used the cross-validated sort from trials before the switch (or the first half of trials if reward did not move) on the reference day (the first day in each day pair) and applied this sort to trials after the switch (or second half) on both the reference day and all trials on the target day (the second day in the pair). To measure drift between days, we computed the across-day circular–circular correlation coefficient between the sequence positions on reference day ‘after’ trials versus target day ‘before’ trials. A minimum of five followed cells in the sequence was required to compute a correlation coefficient.

To quantify the density of sequences, we divided the number of cells with peaks in each 2π / 45 position bin by the total number of place cells for that animal. These density curves were smoothed only for visualization with a one-bin s.d. Gaussian and are shown as the mean across animals per each switch day (Fig. 4 and Extended Data Figs. 4 and 5). We visualized the expected uniform distribution as the fraction of place cells in each animal’s sequence on a given switch day, divided by the number of position bins, averaged across animals.

### Linear mixed-effects models

To quantify neural and behavioral changes across task experience, we used linear mixed-effects models (mixedlm method of statsmodels.formula.api) to account for variance across animals. The fixed effect was usually either the switch index or day-pair index as a continuous variable unless otherwise noted. Random effects were mouse identity. In cases of only one fixed effect term, no corrections for multiple comparisons were made. Random intercepts for each mouse were allowed; we also confirmed that including random slopes did not affect the results. When the dependent variable was reported in fractions of cells, we applied a logit transform to the fractions when a large proportion of the values were near zero. In these cases, an expit transform was applied to the model prediction to plot it with the fractional data points. In most cases, however, we chose to use the original fractions in the model for interpretability, and we confirmed that performing a logit transform on the fractions did not qualitatively change the results and only modestly changed the model fit.

### Analysis of teleport periods

In the sessions for which we imaged the whole teleport period (see ‘2P imaging’), we re-identified place cells including activity during the ‘tunnel’ and part of the ‘jitter’ in the teleport period (see ‘Behavioral training and VR tasks’ for description), and re-categorized remapping types as either TR or RR from this set of place cells. Given that the length of each jitter was different, we only included spatial bins during the jitter that were occupied with fewer than ten trials missing in the session, using spatial bins of 10 cm starting at −50 cm (the start of the tunnel) up to the maximum occupied bin (range, 530–580 cm from the start of the virtual track at 0 cm). We then re-performed the population sequence analysis (see ‘Sequence detection and quantification’), up to the maximum distance occupied across mice (580 cm); note that some bins during the teleport period will include data from fewer animals (Extended Data Fig. 5). To simulate the ‘ideal’ RR remapping destination if RR fields moved by the exact amount that the reward zone moved, we added the circular difference between reward zone starts to the pre-switch peak activity position of each RR cell.

### Analysis of rewarded versus omission trials

Analyses were restricted to trial sets (before or after the reward switch) that had at least three omission trials within the set.

#### Time warp modeling

We first fit five different time warp model types—shift, linear, piecewise one knot, piecewise two knots and piecewise three knots^36^—on the matrix of speed profiles within a trial set (*i* trials × *j* linear position bins × 1, expanded in the third dimension for compatibility with the time warp algorithm). These models apply warping functions of increasing nonlinear complexity to stretch and compress the data on each trial for maximal alignment^36^. We included both rewarded and omission trials in the fitting procedure to find the best alignment across them. Model fit was assessed using the mean squared error between the time-warped speed profile of each trial and the mean across time-warped trials (Extended Data Fig. 8a). The piecewise three knots model most often produced the best fit (lowest mean squared error) across sessions (Extended Data Fig. 8c). We therefore re-fit all sessions with piecewise three knots to ensure that model selection could not influence the results. We then applied the model transform to the deconvolved neural activity matrix for the same set of trials (*i* trials × *j* linear position bins × *n* neurons; excluding neural data at movement speeds of <2 cm s^−1^). For neural analysis in Fig. 6, we then focused on trial sets before the switch in which the reward zone was at position ‘A’ or ‘B’, because the mean rewarded versus omission speed profiles were more similar on these trials than after the switch (Extended Data Fig. 8f) or when the reward was at ‘C’ (as there is less room at the end of the track for running speed to reach its maximum from position ‘C’ on omission trials; see Extended Data Figs. 1 and 8h for examples; see Extended Data Fig. 8 for analyses of trials after the switch and for all reward zones).

#### Reward versus omission index

We compared neural activity on rewarded versus omission trials for RR cells with spatial peaks between the start of the reward zone and the end of the track (under the hypothesis that these cells could receive information about whether reward was received or not on that trial). Using the time warp model-transformed neural activity averaged across rewarded or omission trials within the trial set of interest, we calculated a reward versus omission index for each cell as:

$${\rm{RO}}\; {\rm{index}}=\frac{\sum _{j}\bar{{r}}_{j}-\bar{{o}}_{j}}{\sum _{j}\bar{{r}}_{j}+\bar{{o}}_{j}},$$

where $\bar{{r}}_{j}$ and $\bar{{o}}_{j}$ are the mean activity in position bin *j* averaged across rewarded trials or omission trials, respectively. Reward versus omission indices of 1 indicate an exclusive firing preference for rewarded trials, −1 indicates an exclusive preference for omission trials and 0 indicates equal firing between rewards and omissions. We confirmed that the reward versus omission index calculated from the model-transformed activity was highly correlated with the reward versus omission index calculated from the original activity (Extended Data Fig. 8e). Using a linear mixed-effects model (Extended Data Fig. 8j), we found that there was no significant change in the median reward versus omission index for each animal across days as a result of the model fit or as a result of the original mean squared error between rewarded and omission trials. We therefore combined neurons across days for presentation in Fig. 6e.

### GLM

We implemented a Poisson GLM developed previously^37^ to predict the deconvolved calcium event time series of individual neurons from a set of task and movement variables. All behavioral and neural time series were sampled at ~15.5 Hz, the imaging frame rate.

#### Design matrix

Our design matrix included three task variables and three movement variables (Extended Data Fig. 9a). Task variables were linear track position (from 0 to 450 cm), RR position (from −π to π, centered around the start of each reward zone at 0 radians) and ‘rewarded’, a binary that switches from 0 to 1 when the reward is delivered on each trial and stays at 1 until the end of the trial (stays at 0 on omission trials). Linear track position and RR position were separately expanded into 45 cosine basis functions, one for each 10 cm or ~0.14 radian bin. ‘Rewarded’ was multiplied by the linear position basis functions to yield the interaction between position and whether the animal had been rewarded on a given trial.

Movement variables were running speed and acceleration (smoothed with a five-sample s.d. Gaussian) and smoothed lick count (binary licks per frame smoothed with a two-sample s.d. Gaussian to approximate an instantaneous rate). Each movement variable was separately quantile-transformed to encode the distribution of movement dynamics similarly across animals. The quantiles were expanded with B-spline basis functions using the SplineTranformer method of Python’s sklearn package, with three polynomial degrees and five knots, resulting in seven bases (5 + 3 − 1) per movement variable. Spline choices were made for consistency with previous work^37^.

All predictors were concatenated into a design matrix with 156 total features: 45 position bases + 45 RR position bases + 45 rewarded-by-position bases + 7 speed bases + 7 acceleration bases + 7 licking bases. The features were independently *z*-scored across samples before model fitting on the ‘response matrix’ of deconvolved activity of all cells (samples × neurons).

#### Model fitting and testing

Trial identities were used to group data for training and testing. Individual sessions (including trials both before and after the reward switch) were split into 85% training trials and 15% testing trials, using the GroupShuffleSplit method of Python’s sklearn package with a consistent random seed. Specifically, trials were allocated such that the training trials included 85% of rewarded trials before the switch, 85% of rewarded trials after the switch and 85% of all omission trials. The training trials were further divided for fivefold cross-validation during the fitting procedure. An optimal model was selected according to the deviance explained on the fivefold cross-validation data. We then tested this model on the held-out test data to assess model performance as the fraction deviance explained (FDE):

$${\rm{FDE}}=1-\frac{{\rm{dev}}_{\rm{model}}}{{\rm{dev}}_{\rm{null}}},$$

where dev_model_ is the Poisson deviance of the full model and dev_null_ is the Poisson deviance of a null model that predicts the mean of the neural activity across time samples.

On average, the model’s FDE for all place cells was 0.10 ± 0.19 (mean ± s.d.) (Extended Data Fig. 9b,c), 0.32 ± 0.13 for TR cells, 0.29 ± 0.11 for RR cells (examples in Fig. 7a, b) and 0.29 ± 0.11 for non-RR remapping cells. For analysis of the relative contribution of individual variables, we only included cells with FDE > 0.15 in accordance with previous procedures^37^.

#### Relative contribution of individual variables by model ablation

To assess how much each variable contributed to the model’s ability to predict a given cell’s activity, we performed a model ablation procedure. After fitting the full model, we zeroed the coefficients for each variable and compared the performance of the ablated versus the full model on the cross-validation data. We calculated the reduction in model fit (deviance) of the ablated model relative to the full model, normalized by the full model’s deviance relative to the null model. This is known as ‘fraction explained deviance’^37^, which we term ‘relative contribution’ to distinguish it from FDE. Relative contribution was thus computed as:

$${\rm{Relative}}\; {\rm{contribution}}=\frac{{\rm{dev}}_{\rm{ablated}}-{\rm{dev}}_{\rm{full}}}{{\rm{dev}}_{\rm{null}}-{\rm{dev}}_{\rm{full}}}.$$

Relative contribution was binned by linear track position to visualize the contribution at each position along the track, then averaged across position bins for quantifying the contribution at the population level (Fig. 7d) and identifying the top predictor (variable with the maximum contribution) per cell (Fig. 7e).

### *K*-means clustering and distance score analysis

We applied previously developed factorized *k*-means and distance score algorithms^38,68^ to identify remapping times at the trial-by-trial level. Here, we used the deconvolved activity matrices of each neuron (*i* trials × *j* 10 cm linear position bins), smoothed with a 10 cm s.d. Gaussian. We first scaled activity between 0 and 1 to be comparable across neurons, normalizing each neuron to itself by subtracting its minimum and dividing by the difference between maximum and minimum activity over the session. We then created a population vector for each subpopulation of interest (*i* trials × *j* position bins × *n* neurons), used to create the population correlation matrices shown in Fig. 8 and Extended Data Fig. 10 (see ‘Trial-by-trial similarity matrices’). To identify two discrete clusters in the population activity corresponding to a pre-reward-switch and post-switch ‘map’, we fit a factorized *k*-means model with *k* = 2 and 100 restarts using the open source lvl package^38^. This model factorizes the normalized population activity array *X*_*ijn*_ as:

$${X}_{{ijn}}=\mathop{\sum }\limits_{k=1}^{K}{U}_{i}^{\,(k)}{V}_{{jn}}^{\,(k)},$$

where ${U}_{i}^{\,(k)}$ is an *I* × *K* matrix in which every row is a trial *i* and each column is a one-hot vector encoding the assignment of the trial to each cluster *k*. ${V}_{{jn}}^{\,(k)}$ is a *K* × *J* × *N* array specifying the *J* × *N* (bins × simultaneously imaged neurons) cluster centroid, or population ‘map’, for each cluster *k*.

To determine whether the *k* = 2 model fit was significantly better than could be expected from a session with only one ‘map’, we first cross-validated the model performance using a randomized speckled holdout of 10% of the data over 50 repetitions, then compared the uncentered test *R*^2^ to the test performance on a shuffled dataset. The shuffle was generated by random rotations of the population vector across trials^38^. Sessions in which the real model’s performance at *k* = 2 clusters exceeded that of the shuffle (two-sided Wilcoxon signed-rank test, *P* < 0.05) were accepted for further analysis (*n* = 50 out of 77 sessions for the RR population vector). We also confirmed that results were similar when restricting analysis to sessions best fit by *k* = 2 clusters compared to *k* = 3 or *k* = 4 using a silhouette score approach^68^.

Next, we standardized the cluster labels such that cluster one was always the first cluster appearing in the session (that is, before the reward switch, though we did not restrict the number of trials that could be assigned to cluster one), and cluster two was always the second. We then computed a distance score of how close the population vector activity is to each cluster centroid on each trial^38^, for which a distance score of −1 corresponds to the first cluster in the session and +1 corresponds to the second cluster. The distance score *P* on each trial *i* is computed as:

$${P}_{i}=\frac{\sum _{{jn}}\left(2{X}_{{ijn}}-\left({V}_{{jn}}^{\,\left(1\right)}+{V}_{{jn}}^{\,\left(2\right)}\right)\right)\left({V}_{{jn}}^{\,\left(1\right)}+{V}_{{jn}}^{\,(2)}\right)}{\sum _{{jn}}{\left({V}_{{jn}}^{\,(1)}+{V}_{{jn}}^{\,(2)}\right)}^{2}}.$$

When *P*_*i*_ = 0, the network activity is at the midpoint between clusters, but there are no restrictions on how often *P*_*i*_ can cross this midpoint. To identify an inflection point between sets of trials for which the network activity transitioned from being primarily in cluster one to primarily in cluster two, we fit a sigmoid to the distance score and defined the inflection point of the sigmoid as the ‘remap trial’. This remap trial can be thought of as the point at which the network activity significantly deviates from its pre-switch map.

To identify transitions in behavior, we similarly maximum-normalized the spatially binned lick counts or spatially binned running speed, yielding matrices of *i* trials × *j* position bins × 1. We fit factorized *k*-means models with *k* = 2 to the normalized matrices, computed a distance score and fit a sigmoid to find a remap trial for licking and speed. For subsequent quantification of the difference between neural and behavioral remap trials, sessions were only included if the sigmoidal regression converged for each of the population vector, licking and speed distance scores.

### Reporting summary

Further information on research design is available in the Nature Portfolio Reporting Summary linked to this article.

## Online content

Any methods, additional references, Nature Portfolio reporting summaries, source data, extended data, supplementary information, acknowledgements, peer review information; details of author contributions and competing interests; and statements of data and code availability are available at 10.1038/s41593-025-01985-4.

## Supplementary information

Reporting Summary

## Source data

Source Data Fig. 1 Histology image for Fig. 1b.

Source Data Fig. 1 Statistical source data.

Source Data Fig. 2 Statistical source data.

Source Data Fig. 3 Statistical source data.

Source Data Fig. 4 Statistical source data.

Source Data Fig. 5 Statistical source data.

Source Data Fig. 6 Statistical source data.

Source Data Fig. 7 Statistical source data.

Source Data Fig. 8 Statistical source data.

Source Data Extended Data Fig. 2 Statistical source data.

Source Data Extended Data Fig. 3 Statistical source data.

Source Data Extended Data Fig. 4 Statistical source data.

Source Data Extended Data Fig. 5 Statistical source data.

Source Data Extended Data Fig. 6 Statistical source data.

Source Data Extended Data Fig. 7 Statistical source data.

Source Data Extended Data Fig. 8 Statistical source data.

Source Data Extended Data Fig. 10 Statistical source data.

## Data Availability

Data formatted for use with custom code are available on Figshare (10.25452/figshare.plus.27098065 (ref. ^69^), 10.25452/figshare.plus.27138633 (ref. ^70^)); data in the Neurodata Without Borders format are available on DANDI (10.48324/dandi.001361/0.250406.0045). Source data for specific figure panels are available in the Supplementary Information. Source data are provided with this paper.

## Code availability

Custom Python code for analyzing the data is available at https://github.com/GiocomoLab/Sosa_et_al_2024.
