# Supplementary material for: A flexible hippocampal population code for experience relative to reward
Source: Nat Neurosci. 2025 Jun 11;28(7):1497–509. doi: 10.1038/s41593-025-01985-4 (PMC12229899; doi:10.1038/s41593-025-01985-4)
Supplement: Supplementary file 1 — Reporting Summary [file 41593_2025_1985_MOESM1_ESM.pdf]

nature portfolio

Corresponding author(s): Marielena Sosa, Lisa M. Giocomo

Last updated by author(s): Apr 12, 2025

Reporting Summary

Nature Portfolio wishes to improve the reproducibility of the work that we publish. This form provides structure for consistency and transparency in reporting. For further information on Nature Portfolio policies, see our Editorial Policies and the Editorial Policy Checklist.

Statistics

For all statistical analyses, confirm that the following items are present in the figure legend, table legend, main text, or Methods section.

n/a	Confirmed	
☐	☒	The exact sample size (*n*) for each experimental group/condition, given as a discrete number and unit of measurement
☐	☒	A statement on whether measurements were taken from distinct samples or whether the same sample was measured repeatedly
☐	☒	The statistical test(s) used AND whether they are one- or two-sided<br>*Only common tests should be described solely by name; describe more complex techniques in the Methods section.*
☐	☒	A description of all covariates tested
☐	☒	A description of any assumptions or corrections, such as tests of normality and adjustment for multiple comparisons
☐	☒	A full description of the statistical parameters including central tendency (e.g. means) or other basic estimates (e.g. regression coefficient) AND variation (e.g. standard deviation) or associated estimates of uncertainty (e.g. confidence intervals)
☐	☒	For null hypothesis testing, the test statistic (e.g. *F*, *t*, *r*) with confidence intervals, effect sizes, degrees of freedom and *P* value noted<br>*Give P values as exact values whenever suitable.*
☒	☐	For Bayesian analysis, information on the choice of priors and Markov chain Monte Carlo settings
☒	☐	For hierarchical and complex designs, identification of the appropriate level for tests and full reporting of outcomes
☐	☒	Estimates of effect sizes (e.g. Cohen's *d*, Pearson's *r*), indicating how they were calculated

Our web collection on statistics for biologists contains articles on many of the points above.

Software and code

Policy information about availability of computer code

Data collection	Behavioral data were collected using custom code written for Unity (version 2020.3.19) for virtual reality experiments. Calcium imaging data were collected using Scanbox version 4.7 (https://scanbox.org/).
Data analysis	Custom data analysis code is available at: https://github.com/GiocomoLab/Sosa_et_al_2024. Calcium imaging data were processed using suite2p (https://github.com/MouseLand/suite2p, v0.10.3). The following Python (v3.8.5) packages were used for statistical analyses: pingouin v0.5.0, scipy v1.7.3, astropy v4.0.2, statsmodels v0.12.0, uncertainties v3.1.6, sklearn v1.0.2. The following open source repositories were also used: pycircstat for circular mean tests (https://github.com/circstat/pycircstat), phase-precession for circular-circular correlations (https://github.com/CINPLA/phase-precession), affinewarp for time warp modeling (https://github.com/ahwillia/affinewarp), GLM_Tensorflow_2 for the generalized linear model (https://github.com/HarveyLab/GLM_Tensorflow_2), and lvl for factorized k-means clustering (https://github.com/ahwillia/lvl/tree/master/lvl).

For manuscripts utilizing custom algorithms or software that are central to the research but not yet described in published literature, software must be made available to editors and reviewers. We strongly encourage code deposition in a community repository (e.g. GitHub). See the Nature Portfolio guidelines for submitting code & software for further information.

Data

Policy information about availability of data

All manuscripts must include a data availability statement. This statement should provide the following information, where applicable:

- Accession codes, unique identifiers, or web links for publicly available datasets
- A description of any restrictions on data availability
- For clinical datasets or third party data, please ensure that the statement adheres to our policy

Data required to reproduce the main results of the paper are on Figshare (DOI: 10.25452/figshare.plus.27098065 and 10.25452/figshare.plus.27138633), as well as on DANDI in Neurodata Without Borders format at https://doi.org/10.48324/dandi.001361/0.250406.0045. Data are available under a CC-BY-4.0 license.

Research involving human participants, their data, or biological material

Policy information about studies with human participants or human data. See also policy information about sex, gender (identity/presentation), and sexual orientation and race, ethnicity and racism.

Reporting on sex and gender	No human data was used for this study.
Reporting on race, ethnicity, or other socially relevant groupings	No human data was used for this study.
Population characteristics	No human data was used for this study.
Recruitment	No human data was used for this study.
Ethics oversight	No human data was used for this study.

Note that full information on the approval of the study protocol must also be provided in the manuscript.

Field-specific reporting

Please select the one below that is the best fit for your research. If you are not sure, read the appropriate sections before making your selection.

☒ Life sciences ☐ Behavioural & social sciences ☐ Ecological, evolutionary & environmental sciences

For a reference copy of the document with all sections, see nature.com/documents/nr-reporting-summary-flat.pdf

Life sciences study design

All studies must disclose on these points even when the disclosure is negative.

Sample size	Depending on the analysis in question, sample size was determined by the number of mice, number of cells, or number of recording sessions. Sample size and the type of data used to determine it are reported in the manuscript. The sample sizes were not calculated by power analyses; however, our sample sizes are comparable or higher than those previously used in the literature (e.g. Danielson et al., 2016). A total of 14 mice are included in the manuscript.
Data exclusions	Cells were excluded from analyses only if they did not meet pre-established criteria as "place cells", defined as having greater spatial information in a given trial set than 95% of shuffles across cells within a mouse. This exclusion ensures that included cells provide reliable information about spatial position in the task.
Replication	Replication was verified by conducting the same experiment in different animals and across different days. An initial cohort of n=7 mice was used for the main "switch" task reported, and all key results were replicated in an additional n=4 mice (total n=11 mice reported for this task).
Randomization	The sequence of reward zone switches was psuedo-randomized and counterbalanced across mice included in the switch task. Mice were also randomly selected to experience the switch task (n=11 mice) versus a "fixed condition" task in which the reward zone was not moved (n=3).
Blinding	Blinding was not necessary as there were no "treatment" groups.

Reporting for specific materials, systems and methods

We require information from authors about some types of materials, experimental systems and methods used in many studies. Here, indicate whether each material, system or method listed is relevant to your study. If you are not sure if a list item applies to your research, read the appropriate section before selecting a response.

Materials & experimental systems

n/a	Involved in the study
☒	☐ Antibodies
☒	☐ Eukaryotic cell lines
☒	☐ Palaeontology and archaeology
☐	☒ Animals and other organisms
☒	☐ Clinical data
☒	☐ Dual use research of concern
☒	☐ Plants

Methods

n/a	Involved in the study
☒	☐ ChIP-seq
☒	☐ Flow cytometry
☒	☐ MRI-based neuroimaging

Animals and other research organisms

Policy information about studies involving animals; ARRIVE guidelines recommended for reporting animal research, and Sex and Gender in Research

Laboratory animals	Male and female (n=7 male, 7 female) C57BL/6J mice were acquired from Jackson Laboratory. Mice were housed in a transparent cage (Innovive) in groups of five same-sex littermates prior to surgery, with access to an in-cage running wheel for at least 4 weeks. After surgical implantation, mice were housed in groups of 1-3 same-sex littermates, with all mice per cage implanted. All mice were kept on a 12-h light–dark schedule, with experiments conducted during the light phase, and housed at ~22 degrees C and 40-45% humidity. Mice were between ~2.5 to ~4.5 months of age at the time of surgery (weighing 18–31 g). Before surgery, animals had ad libitum access to food and water, and ad libitum access to food throughout the experiment.
Wild animals	This study did not involve wild animals.
Reporting on sex	Equal numbers of male and female mice were used in the study, pseudo-randomized to be balanced across task conditions. No overt differences in hippocampal coding were observed between sexes in initial analyses; as such comparison between sexes is not reported.
Field-collected samples	This study did not involve samples collected from the field.
Ethics oversight	All procedures were approved by the Institutional Animal Care and Use Committee at Stanford University School of Medicine

Note that full information on the approval of the study protocol must also be provided in the manuscript.

Plants

Seed stocks	*Report on the source of all seed stocks or other plant material used. If applicable, state the seed stock centre and catalogue number. If plant specimens were collected from the field, describe the collection location, date and sampling procedures.*
Novel plant genotypes	*Describe the methods by which all novel plant genotypes were produced. This includes those generated by transgenic approaches, gene editing, chemical/radiation-based mutagenesis and hybridization. For transgenic lines, describe the transformation method, the number of independent lines analyzed and the generation upon which experiments were performed. For gene-edited lines, describe the editor used, the endogenous sequence targeted for editing, the targeting guide RNA sequence (if applicable) and how the editor was applied.*
Authentication	*Describe any authentication procedures for each seed stock used or novel genotype generated. Describe any experiments used to assess the effect of a mutation and, where applicable, how potential secondary effects (e.g. second site T-DNA insertions, mosiacism, off-target gene editing) were examined.*
