# Supplementary figures and images for: A flexible hippocampal population code for experience relative to reward
Source: Nat Neurosci. 2025 Jun 11;28(7):1497–509. doi: 10.1038/s41593-025-01985-4 (PMC12229899; doi:10.1038/s41593-025-01985-4)

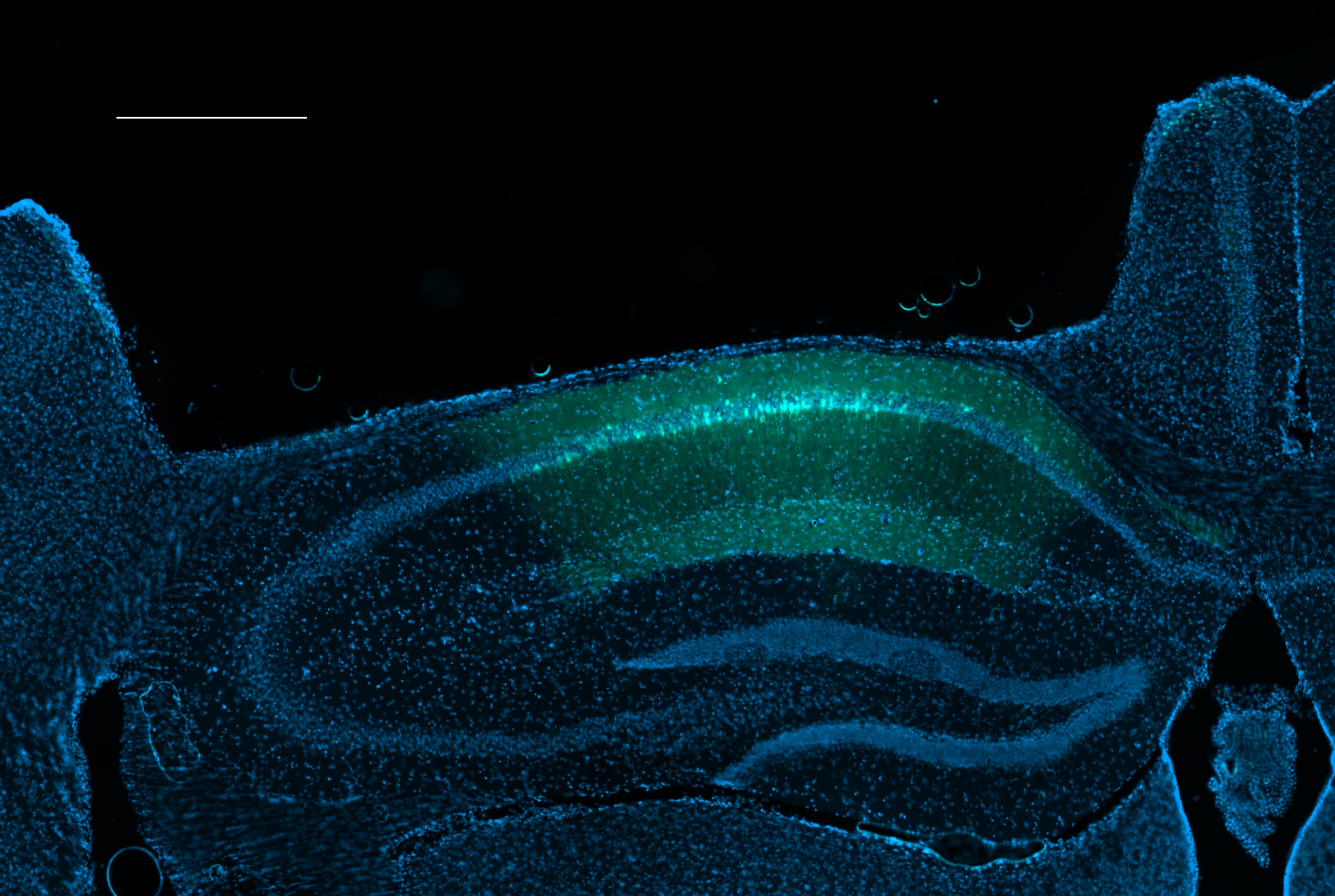

Supplement: Supplementary file 2 — Histology image for Fig. 1b. [file 41593_2025_1985_MOESM2_ESM.jpg]
